# Informed Feature-Based Molecular Networking as a Complementary Approach to Identify Bioactive Phyllobilins in Senescent Plant Extracts

**DOI:** 10.1021/acsomega.5c10395

**Published:** 2025-12-11

**Authors:** Christian A. Elvert, Johanna K. S. Lückenbach, Simone Moser, Fabian Hammerle, Cornelia A. Karg

**Affiliations:** † Center for Molecular Biosciences, 27255 University of Innsbruck, Innrain 80/82, A-6020 Innsbruck, Austria; ‡ Department of Pharmacognosy, Institute of Pharmacy, 27255 University of Innsbruck, Innrain 80/82, A-6020 Innsbruck, Austria; § Institute of Medical Biochemistry, Medical University of Innsbruck, Innrain 80/82, A-6020 Innsbruck, Austria

## Abstract

Phyllobilins are chlorophyll-derived metabolites that represent a distinct class of bioactive natural products. Although widespread in nature, they remain largely underexplored in phytochemical research. In this study, we present a workflow for the dereplication and identification of phyllobilins in complex plant extracts. The approach combines RP-MPLC fractionation, UHPLC-VWD-HRMS^2^ analysis, antioxidant activity testing, and data analysis using feature-based molecular networking. Methanolic extracts of senescent *Echinacea purpurea* leaves, known to be rich in structurally diverse chlorophyll metabolites, were used to generate experimental MS^2^ spectra for a set of phylloxanthobilins, which were subsequently uploaded to the GNPS community database. The workflow was then applied to senescent *Salvia officinalis* leaves, a pharmaceutically important plant with an unknown phyllobilin profile, leading to the annotation of several phylloxanthobilins. One metabolite featuring a malonic acid ester moiety was isolated and structurally confirmed using 1D- and 2D-NMR spectroscopy. Future applications of this workflow may enable the systematic annotation and prioritization of previously overlooked phyllobilins across diverse plant species, addressing a critical gap in the analytical profiling of bioactive plant extracts. By leveraging public MS^2^ spectral libraries and network-based data analysis, it offers a scalable tool to address a long-standing blind spot of natural product research.

## Graphical abstract

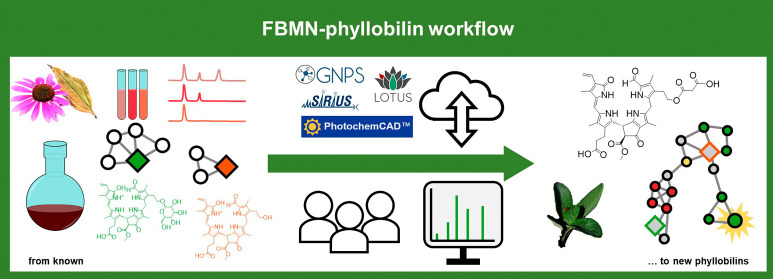

## Introduction

Phyllobilins (PBs) are linear tetrapyrroles formed via the PaO/phyllobilin pathway, named after pheophorbide a oxygenase (PaO), the enzyme responsible for cleaving the chlorophyll macrocycle.^1^ This reaction yields the red chlorophyll catabolite (RCC),^2,3^ which is further converted into primary phyllolumibilins and subsequently modified into diverse derivatives (*modified* phyllolumibilins) through side-chain modifications at rings A and D (Chart 1). These compounds are transported to the vacuole, where the acidic pH induces a nonenzymatic tautomerization into colorless phylloleucobilins (PleBs), the only nonenzymatic step identified in the pathway to date.^4,5^ Still, there are steps of the pathway that are not completely understood, such as the oxidation process of the PleBs resulting in yellow products, the phylloxanthobilins (PxBs), and the oxidation of PxBs to the pink phylloroseobilins (PrBs) (Chart 1).^6^ Most known PBs feature an α-formylpyrrole at ring A, classified as type I PBs, while type II PBs, bearing a pyrrolinone, arise via CYP450-catalyzed deformylation.^7^ Up to date, over 70 different structures have been discovered across various plant families, including Monocotyledonae and Dicotyledonae;^8^ however, the full extent of their biodiversity remains far from fully elucidated.

**Chart 1:**
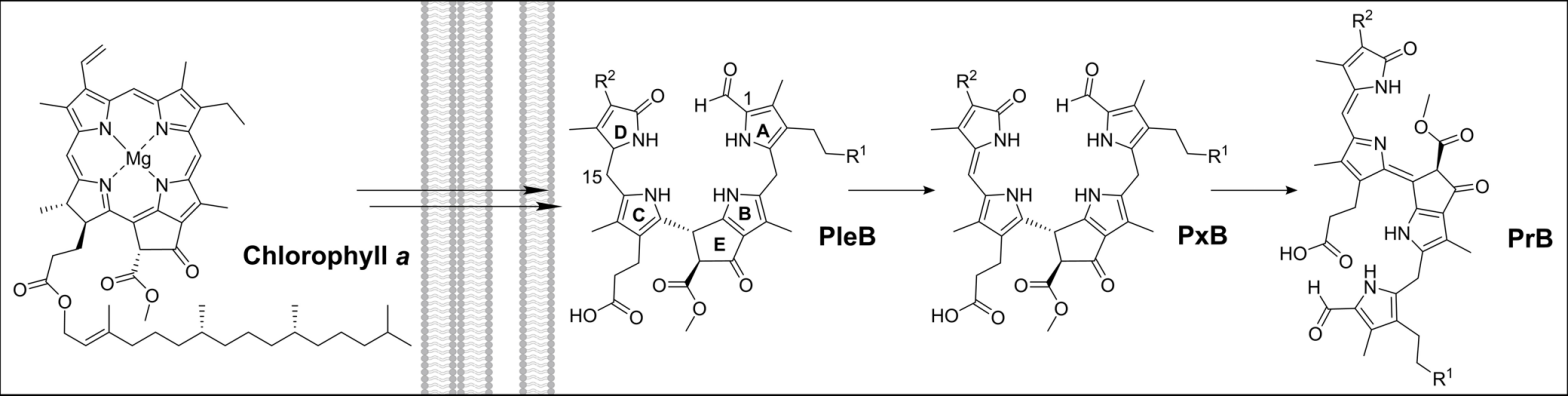
Chemical Outline of the Chlorophyll Breakdown Process Featuring Late-Stage Phyllobilins (PleB, PxB, and PrB)^a^

a Structural diversity arises among others from different modifications at ring A (R^1^) and D (R^2^).

PBs, long regarded as detoxification byproducts of chlorophyll breakdown, are now recognized as widespread and potentially bioactive plant metabolites, as recent studies have uncovered pharmacologically relevant bioactivities.^7^ In *Echinacea purpurea*, six structurally distinct PxBs were identified, all displaying antioxidant properties *in vitro*, with selected compounds also showing reactive oxygen species (ROS)-scavenging activity in cellular assays.^9^ Additionally, PBs inhibited IDO-1 activity in human immune cells and demonstrated anti-inflammatory effects.^10,11^ A PxB from *Cercidiphyllum japonicum* (also found in *E. purpurea* ^9^) inhibited cancer cell proliferation at low micromolar concentrations,^12^ and the same PxB as well as a PrB were found to inhibit cancer cell migration, with the cytoskeletal protein actin identified as the first human molecular target for this compound class.^13^ PBs are highly abundant, particularly in aged or senescent plant material. Quantitative analyses have documented levels of up to 25 μg PBs per gram of stored broccoli,^14^ and as much as 100 μg of a single PxB in one cup of nettle tea.^11^ Given their widespread presence and measurable dietary exposure, PBs likely represent a biologically relevant group of plant-derived metabolites.

Identification of PBs is usually performed using techniques based on their characteristic absorbance properties.^8^ Since they exhibit distinct UV/vis absorption maxima, high-performance liquid chromatography with diode-array detection (HPLC-DAD) has been a crucial method for their identification in crude plant extracts. Especially the phyllobilin type can be determined based on the absorption properties; for instance, PxBs show a characteristic absorption maximum at around 420 nm. These UV-based signatures have been summarized very recently in a comprehensive UV database (https://www.photochemcad.com).^8^

Nevertheless, PB identification and isolation remain challenging, and levels can vary widely even among samples from the same plant species; they are sensitive to light and can degrade at elevated temperatures. These factors complicate not only the discovery and structural elucidation via NMR but also the evaluation of biological activity. In this context, mass spectrometry (MS) has become an indispensable tool for PB research, particularly for elucidating structural features and modification patterns of the PB core. The gas-phase behavior of PBs is well characterized, with consistent fragmentation patterns such as the loss of water, methanol, ring A, and ring D.^15^ A publicly available MS^2^-based PB database containing 16 compounds from *Arabidopsis thaliana* has already enabled successful identification of chlorophyll catabolites in other plant matrices.^16^ Building on this foundation, other groups have also effectively applied MS for analyses of PBs.^17,18^

Despite growing evidence of their bioactivity and their ubiquity as plant metabolites in deciduous plants as well as constituents of plant-based food,^19^ PBs still remain largely overlooked in natural product research and are absent from major natural product databases such as LOTUS,^20^ which considerably hampers their broader recognition and identification.

In recent years, untargeted MS-based metabolomics has become an integral part of natural product research, enabling both the rapid dereplication of complex extracts and the identification of links between bioactivity and chemical constituents. While the output of such analyses is initially vast and challenging to interpret, methods like molecular networking^21^ and its successor, feature-based molecular networking (FBMN),^22^ have facilitated more accessible and systematic data exploration. Both approaches group constituents based on MS^2^ spectral similarity, typically from (U)­HPLC-HRMS^2^ data, into “molecular families”, compounds with minor structural differences that often share a common chemotaxonomic origin. Freeware such as mzmine^23^ and MSDIAL^24^ allows reproducible preprocessing, while the GNPS web platform (https://gnps.ucsd.edu) supports network creation, annotation, and integration of quantitative data. Established protocols for generation^25^ and statistical evaluation^26^ further enhance reproducibility and interpretability. Literature-based dereplication helps redirect efforts toward the discovery of novel bioactive compounds. An enhanced workflow, ion identity molecular networking (IIMN),^27^ links multiple ion species of the same compound that otherwise appear as separate, unconnected nodes. This prevents the artificial disruption of molecular families and improves propagation of database annotations across chemical space. FBMN and IIMN have found wide application, from the annotation of photoactive pigments in basidiomycetes^28,29^ to the discovery of alkaloids^30^ and mycosporine-like amino acids^31^ in marine organisms, and even explorations of chemical diversity aboard the International Space Station (ISS).^32^ The annotation efficiency of these tools, however, depends heavily on the availability and coverage of MS^2^ spectral databases. This poses a particular challenge for PBs, which are not represented in the community GNPS library or in many of the compound databases accessed by downstream software such as SIRIUS,^33^ including Coconut^34^ and SuperNatural.^35^ In addition, no freely accessible experimental MS^2^ library currently exists for chlorophyll metabolites.

To address the analytical blind spot surrounding PBs in plant metabolomics, we developed an integrated workflow for their dereplication and identification in complex natural extracts. This approach combines RP-MPLC fractionation, antioxidant activity testing, and UHPLC-VWD-HRMS^2^ analysis, followed by data visualization using FBMN. Methanolic extracts of senescent *E. purpurea* leaves, rich in structurally diverse, well-characterized PBs, were used to generate high-quality MS^2^ spectra, which were uploaded to the GNPS public database. This spectral resource enabled the targeted screening of *Salvia officinalis*, a well-known medicinal plant not previously reported to contain PBs, revealing new candidates. One PB featuring a malonic acid ester, previously found in *Ocimum basilicum*, was isolated and structurally confirmed via 1D- and 2D-NMR.

## Experimental Section

### Preparation of Plant Extracts

Senescent leaves of *E. purpurea* and *S. officinalis* were used for the preparation of extracts. Leaves were collected at the botanical gardens of Munich and Innsbruck (voucher specimen *S. officinalis*: IB 117058). Leaves were freeze-dried and pulverized with a grinder. For the *E. purpurea* extract, 145 g of leaves, and for the *S. officinalis* extract 93 g of leaves were used. Afterward, the powdered leaves were ultrasonicated with methanol three times. The combined methanolic extracts were evaporated and then lyophilized to obtain 4.5 g of *E. purpurea* and 3.5 g of *S. officinalis* extract.

### Fractionation of Plant Extracts

1.25 g of the extract was suspended with 20% (v/v) of methanol and subjected again to ultrasonic treatment for 10 min and then centrifuged at 2500*g* for 5 min. The supernatant was applied to a Strata C18 solid-phase extraction cartridge (Phenomenex, Aschaffenburg, Germany). The column was washed with water, and the extract was eluted with methanol. Following evaporation, the residue was fractionated using a Reveleris X2 flash chromatography system with an integrated UV/vis and ELS detector on a Büchi FlashPure C18 cartridge (40 μm, irregular, 40 g) with phosphate buffer (*c* = 50 or 100 mM, pH = 7) (A) and methanol (B) as mobile phases. Fractions were collected using a linear gradient with increasing methanol concentrations: 7–14% B for F1, 14–21% B for F2, 21–28% B for F3, 28–35% B for F4, 35–41% B for F5, 41–48% B for F6, 48–55% B for F7, 55–62% B for F8, 62–68% B for F9, 68–75% B for F10, 75–82% B for F11, 82–89% B for F12, and 89–92% B for F13. Chromatograms of the fractions and additional chromatographic parameters can be found in the Supporting Information (Figure S1, Text S1). The fractions were desalted using a C18 solid-phase extraction cartridge, dried, and dissolved with DMSO to achieve a final concentration of either 5 or 30 mg/mL, depending on the yield.

### Data Acquisition

The UHPLC analysis of the extracts (*c* = 5 or 30 mg/mL in HPLC-grade DMSO) was performed on a Vanquish system (Thermo Scientific, Waltham, Massachusetts, USA) consisting of a binary pump, an autosampler, a column oven, and a variable wavelength detector connected to a Thermo Scientific Exploris 120 Orbitrap HRMS unit. Separation was carried out on a Waters ACQUITY UPLC BEH C18 column (2.1 mm × 50 mm, particle size = 1.7 μm) (Waters Corporation, Milford, Massachusetts, USA) protected by a Phenomenex SecurityGuard ULTRA guard cartridge system (i.e., a UHPLC C18 precolumn) (Phenomenex, Aschaffenburg, Germany). The mobile phase comprised ammonium formate (*c* = 10 mM) in water with 0.1% formic acid (A) and acetonitrile (B). The applied gradient was as follows: 0 min, 10% B; 3 min 20% B; 10 min, 20% B; 11 min, 25% B; 17 min, 30% B; 20 min, 95% B; 26 min, 95% B. Finally, the column was re-equilibrated with the original solvent composition for 12 min. The flow rate, column oven temperature, autosampler temperature, and injection volume were adjusted to 0.3 mL/min, 35 °C, 20 °C, and 2 μL, respectively. The detection wavelengths were set to 320 and 420 nm, the data collection rate to 1.0 Hz, the response time to 5.0 s, and the peak width to 0.5 min.

The system was controlled by Thermo Scientific Xcalibur 4.4 software. Calibration of the mass analyzer was done via the Thermo Scientific proprietary calibration mix and the respective automatic calibration function. Detailed descriptions for the mass spectrometric parameters as well as the data processing can be found in the Supporting Information (Text S2).

### Feature-Based Molecular Network Generation

A molecular network was created with the FBMN workflow on GNPS.^22^ The jobs are publicly available at: https://gnps.ucsd.edu/ProteoSAFe/status.jsp?task=b334a2b080d34b5c9f484bd787507a22 for *E. purpurea* and https://gnps.ucsd.edu/ProteoSAFe/status.jsp?task=cce76452248d42b59ded3c5e85c947b2 for *S. officinalis*. Quantification tables, .mgf files, and the in-house phyllobilin database can be accessed via zenodo: 10.5281/zenodo.17243849. A detailed description about the network generation, network parameters, and additional network filters can be found in the Supporting Information (Text S3).

### Isolation and Structure Elucidation of a Phylloxanthobilin from *S. officinalis* Leaves

2 g of the dried methanolic extract of *S. officinalis* was applied to a C18 solid-phase extraction cartridge. The extract was eluted with methanol and evaporated until dry. The residue was dissolved in a mixture of acetonitrile and aqueous ammonium formate (*c* = 10 mM, 20/80 v/v) and purified with a preparative HPLC system to obtain a fraction with a pure PxB (*Sao*-PxB). Detailed information about the isolation process, as well as spectroscopic data can be found in the Supporting Information (Text S4 and S5).

### Assessing the Bioactivity Potential of Plant Fractions

The antioxidative potential of the fractions of *E. purpurea* and *S. officinalis* extracts were determined using a ferric reducing antioxidant power (FRAP) assay by following the protocol from Benzie and Strain with minor adaptations.^36^ A detailed description can be found in the Supporting Information (Text S6).

Intracellular ROS in human keratinocytes were assessed following the protocol from a previous study.^10^ A detailed description can be found in the Supporting Information (Text S7).

## Results and Discussion

### Study Workflow

In this study, we developed an MS^2^-based workflow for the identification of PBs in complex plant extracts (Figure 1). Methanolic extracts of senescent *E. purpurea* leaves were fractionated and analyzed by UHPLC-VWD-ESI-HRMS^2^ to generate reference MS^2^ spectra. Known PBs (*Ep*-PxBs) were annotated after data visualization and evaluation with FBMN, integrating MS^2^, chromatographic, and UV/vis information. Representative spectra were added to the GNPS community library and used to profile PBs in *S. officinalis*, a medicinal plant not previously investigated for this compound class. Using the same extraction, fractionation, and analysis workflow, a second FBMN was generated. Incorporation of bioactivity data from FRAP and ROS assays enabled the identification of a bioactive PB cluster. A previously unreported PB from *S. officinalis* was isolated and structurally confirmed by NMR, demonstrating the potential of this streamlined approach for natural product research.

**Figure 1:**
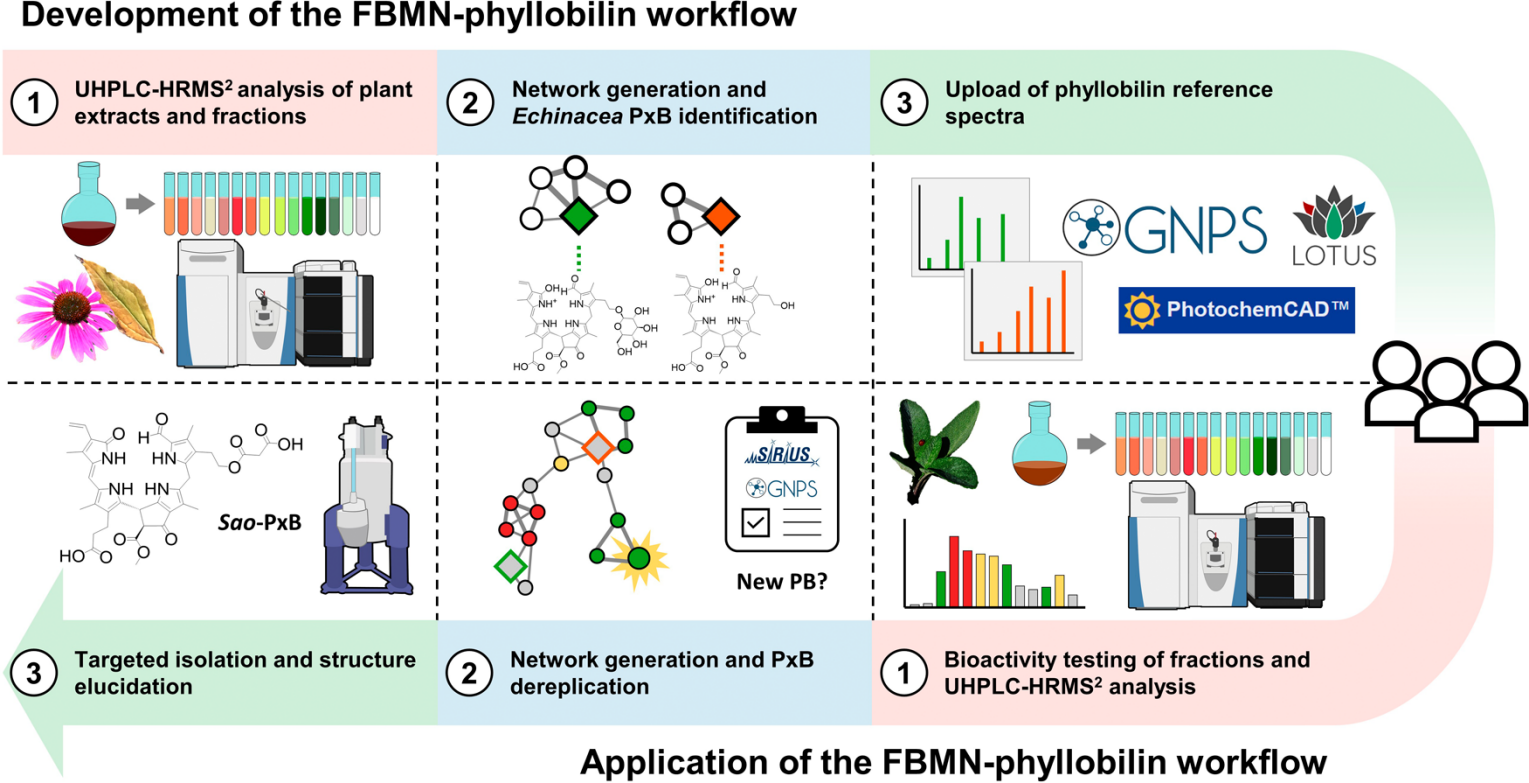
Overview of the workflow: from the development to its application. Illustration includes elements from NIAID NIH BioArt Source (bioart.niaid.nih.gov/bioart/392; bioart.niaid.nih.gov/bioart/286).

### Informed FBMN of *E. purpurea* Extracts: Spotting *Ep*-PxBs

*E. purpurea* is a widely used medicinal plant approved by the European Medicines Agency (EMA) for the well-established use in the short-term treatment and prevention of the common cold.^37^ Its extracts contain diverse phytochemicals, including alkylamides and caffeic acid derivatives.^38^ In our lab, we have already specifically investigated *E. purpurea* leaf extracts for the presence of PBs that had long been overlooked in phytochemical research, despite their potential as bioactive natural products.^9^ In total, six diverse PxBs could be identified and were designated with decreasing polarity (*Ep*-PxB-1 to *Ep*-PxB-6) (for structures, see Figure S2).^9^ In the mentioned study, PBs have been effectively identified in raw plant extracts based on their characteristic UV/vis absorption spectra observed during analytical HPLC runs.^9^ These UV-based approaches have laid an essential foundation for PB research, offering a reliable and structurally informative detection method thanks to the distinct optical properties of PBs.^8^ However, as the complexity of extract matrices increases and interest grows in detecting also less abundant or structurally diverse PBs, there is growing value in complementing UV/vis detection with additional analytical tools. To this end, *E. purpurea* was selected as a model system for the development of a comprehensive workflow that integrates HRMS^2^ analysis with molecular networking visualization, while still incorporating UV/vis absorption data.

An extract of senescent *E. purpurea* leaves was separated into 13 fractions using a C18 column. UHPLC-HRMS^2^ analysis with an optimized gradient for the detection of PBs resulted in a comprehensive feature-based molecular network containing 4378 nodes, including 1538 singletons, and 7501 edges (Figure S3).

For the initial network analysis, an absorbance-based filter was implemented to highlight nodes exhibiting an UV/vis absorption at 420 nm above a certain threshold (Figure S4). This wavelength was selected because all currently known PxBs share a characteristic absorption maximum around 420 nm.^8^ This absorbance-based filter served as visual highlighting tool to facilitate the rapid identification of PB clusters. Importantly, PBs with atypical or shifted UV/vis spectra would still be expected to share the characteristic tetrapyrrolic core and thus exhibit similar MS^2^ fragmentation patterns. Therefore, they would likely appear in the same clusters even if they do not meet the optical highlighting criterion. This approach considerably reduced the number of potentially PB-containing clusters. However, unambiguous identification remains challenging due to the presence of different compounds exhibiting similar UV absorption patterns and the potential coelution of chemically unrelated substances.

For the identification of previously reported *Ep*-PxBs, an in-house MS^1^-based PB database was used within mzmine (Text S3). Preliminary annotations were considered credible if the corresponding node also exhibited an absorbance at 420 nm above a defined threshold. To further examine each annotated node, MS^2^ spectra were analyzed and compared with reference data from the literature.^9,39,40^

To highlight the importance of incorporating reference spectra of PBs in publicly available spectral libraries, we attempted to annotate *Ep*-PxBs in the dataset using various chemoinformatic tools designed for natural product classification. First, taxonomically informed metabolite annotations were created with the R-based script TIMA,^41^ which processes MS^1^ data and matches features against entries in natural product databases such as LOTUS.^20^ TIMA reranks candidate structures based on taxonomy scores, favoring metabolites that are more likely to occur in the plant species under investigation or in closely related ones. In parallel, compound class predictions based on MS^2^ spectra were conducted using CANOPUS within SIRIUS software.^42^ This tool uses deep learning to deduce structure-related classes. The resulting annotations for all *Ep*-PxB nodes were further categorized using the ClassyFire chemotaxonomy (Table S1).^43^ The TIMA script only returned unrelated candidate structures, demonstrating the current underrepresentation of PBs in natural product databases. CANOPUS, which bypasses database dependency by using neural network models trained on MS^2^ fingerprints, performed better; however, only 22 out of 28 nodes from both PB-containing clusters were correctly classified as “Tetrapyrroles and derivatives” at the ClassyFire class level. Overall, confidence scores for SIRIUS predictions were low, which further underscores the need for curated MS^2^ reference spectra of PBs to enable accurate and efficient, automated annotation workflows and dereplication strategies in plant metabolomics.

Based on an in-house MS^1^-based PB database, several nodes corresponding to *Ep*-PxB-1 to *Ep*-PxB-5 were localized within a single cluster, whereas nodes matching *Ep*-PxB-6 were found in a separate cluster (Figure 2A). Multiple nodes with equal *m*/*z* values are likely to represent isomeric forms, since stereoisomers with the same mass and similar MS^2^ fragmentation patterns can still be distinguished in FBMN as long as they differ in retention time. In theory, a maximum of four stereoisomeric nodes per compound could be expected: two arising from cis–trans isomerism of the double bond at C15 ^40,44^ and two resulting from epimerization at the C8^2^ position.^45^ Consistent with this expectation, isolated *Ep*-PxB-6 produced multiple chromatographic peaks in the extracted ion chromatogram supporting the interpretation that the corresponding nodes reflect isomeric forms (Figure S12). However, more than four nodes were observed for both *Ep*-PxB-5 and *Ep*-PxB-6, all exhibiting identical masses and similar MS^2^ spectra. Structurally, *Ep*-PxB-4 and *Ep*-PxB-5 differ only at ring A by the additional malonyl ester group at the glucose unit of *Ep*-PxB-4. Therefore, it is plausible that *Ep*-PxB-5 may result from in-source fragmentation of *Ep*-PxB-4 by ester bond cleavage during electrospray ionization (Figure 2B). This may also explain the appearance of additional nodes corresponding to *Ep*-PxB-6, which could be, e.g., the result of hydrolysis of the β-glucoside moiety of *Ep*-PxB-4 or *Ep*-PxB-5, as can be seen by the XIC traces of *Ep*-PxB-6, which overlay with the XIC chromatograms of the other PxBs (Figure 2B). An alternative explanation for the appearance is related to the fractionation process. Broader chromatographic peaks were observed in fractions containing high concentrations of these compounds, potentially leading to the generation of multiple features from the same analyte in the molecular network.

**Figure 2:**
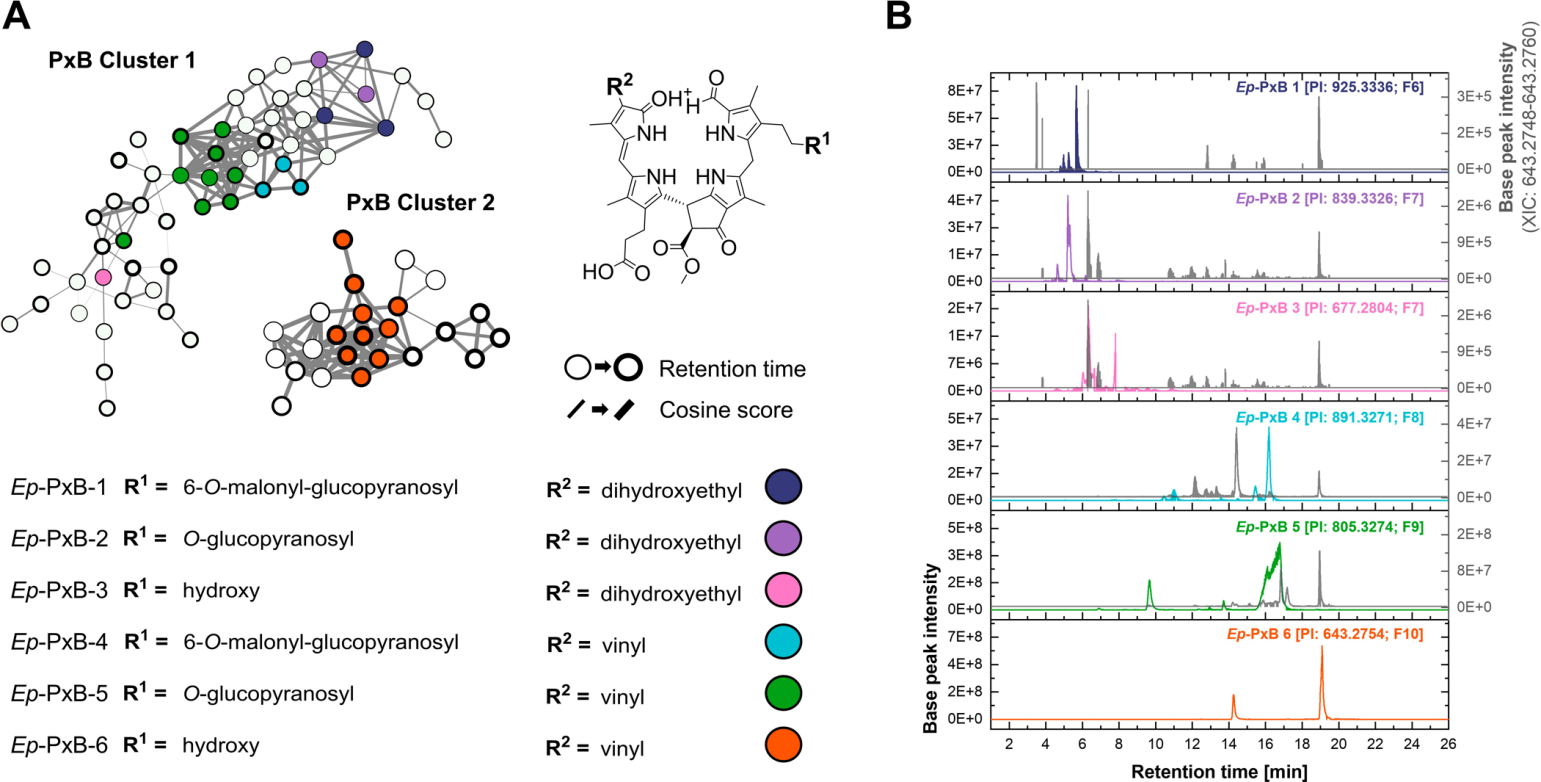
(A) PxB clusters in the *Echinacea* network. Colored nodes represent *Ep*-PxB-1 to *Ep*-PxB-6 ([M + H]^+^). Node border thickness increases with retention time. (B) Extracted ion chromatograms (XICs) of the six identified *Echinacea* phylloxanthobilins (*Ep*-PxB-1 to *Ep*-PxB-6), each plotted using a 1 ppm mass window centered on the mass of the respective precursor ion. To illustrate that fragment ions corresponding to *Ep*-PxB-6 (orange, *m*/*z* 643.2754) can also arise from other phylloxanthobilins during ionization, its XIC trace (gray) is overlaid in all panels. Retention times are given in minutes, and base peak intensities are shown on the *y*-axes.

The structural similarity of the different PxBs is also evident in their location within the molecular network cluster. Thick edges between nodes indicate high spectral similarity, as represented by high cosine scores derived from MS^2^ comparisons. For example, nodes representing *Ep*-PxB-4 and *Ep*-PxB-5 are directly connected by a thick edge (cosine score = 0.9). A similar pattern is observed for *Ep*-PxB-1 and *Ep*-PxB-2. In addition to the MS^2^ similarity, chromatographic retention time data were also implemented in the network visualization. Node borders were linked to retention time on a C18 reversed-phase column: Thick borders indicate later retention times and thus greater hydrophobicity. Accordingly, nodes representing *Ep*-PxB-1 to *Ep*-PxB-3 showed thin borders, reflecting earlier elution and higher polarity, while *Ep*-PxB-4 to *Ep*-PxB-6 showed thicker borders, implying a more apolar character. Finally, the most intense MS^2^ spectra for each *Ep*-PxB node were uploaded to the GNPS spectral library (Table S6).

### Informed FBMN of *S. officinalis* Extracts: Characterization of PB Clusters

*S. officinalis* was chosen for a proof-of-concept study, as there are no reported studies on its PB content or profile. Furthermore, *S. officinalis* extracts are monographed by the EMA for their traditional use in relieving inflammation of the mouth or the throat,^46^ and different studies have demonstrated their anti-inflammatory and antioxidant properties.^47^ These bioactivities have also been ascribed to PBs in other plants.^7^ Although phenolics, flavonoids, and terpenoids are often mentioned as key active constituents of *S. officinalis*,^48^ the potential contribution of PBs to its pharmacological properties remains uninvestigated.

To explore the PB profile of senescent *S. officinalis* leaves, an FBMN was generated from 13 polarity-based fractions that comprised 6640 individual nodes, including 2408 singletons, and 10,832 edges (Figure S5).

The same UV/vis-based filter, highlighting nodes with absorption at 420 nm, was applied to the *S. officinalis* network to facilitate the detection of potential PB clusters (Figure S6). Compared to the *E. purpurea* data set, only a limited number of clusters contained UV/vis active nodes.

With reference spectra now available in the GNPS spectral library, all MS^2^ spectra in the *S. officinalis* network were screened against the uploaded PB reference entries. This query resulted in the annotation of three nodes corresponding to isomers of *Ep*-PxB-6 (Figure S8). These nodes were located within a cluster of 75 nodes and 219 edges, many of which were also assigned a positive 420 nm filter variable, which supported the classification of the cluster as a potential PB molecular family. In order to improve the annotation hit rate, despite the limited number of available reference spectra, we additionally applied the GNPS analog search tool. Using this approach, even more features could be annotated as potential PBs within the same cluster (Figures S7 and S8). Several nodes were tentatively assigned as analogs of *Ep*-PxB-6, which is relatively simple in structure and features a core commonly found in other PBs. Two other features likely corresponded to derivatives of *Ep*-PxB-4 and *Ep*-PxB-5, although precise structural suggestions could not be made.

Once again, we applied the chemoinformatic annotation tools TIMA and CANOPUS to all nodes within the putative PB cluster. This allowed a comparison between the *in silico* prediction and the GNPS analog-based spectral matching. A comprehensive list of annotation results is provided in Table S2. As expected, TIMA did not yield any clear indications of potential PBs within the cluster. In contrast, CANOPUS successfully classified the majority of the nodes as “Tetrapyrroles and derivatives”, thus reflecting the core skeleton of PBs and supporting their correct assignment.

A bioactivity-guided network analysis was conducted to further characterize the PB cluster and identify potentially novel bioactive compounds. Given that previous studies have consistently reported strong antioxidant properties for PBs,^7,9–11,19^ we evaluated the antioxidant potential of all 13 *S. officinalis* fractions with two complementary assays: a chemical-based FRAP assay and an intracellular ROS scavenging assay, since antioxidant activity is influenced by cellular uptake.^49^ The results of both assays were then integrated into the molecular network, allowing visualization and prioritization of bioactive clusters and individual nodes. The antioxidative potential of the *S. officinalis* fractions, based on the results from the FRAP assay, were ranked and visualized using an empirical color-coding system: gray (inactive), green (active), yellow (very active), and red (highly active). Fractions F1, F2, F9, F10, and F13 were considered inactive; F3, F8, and F11 showed moderate activity; F6, F7, and F12 were classified as very active; and F4 and F5 exhibited the highest antioxidant activity. This bioactivity-based color scheme was integrated into the molecular network (Figure 3 and Figure S9), where each node contains a pie chart whose segments correspond to the peak intensity distribution across fractions and are colored according to the respective bioactivity classification. Based on this informed version of the network, one cluster was prioritized for further analysis because many of its nodes were associated with the most active fractions. Annotation of this cluster using CANOPUS (Figure S9 and Table S3) while applying the NPClassifier^50^ as well as the ClassyFire^43^ chemotaxonomies suggested that many of these nodes belong to the terpenoid family, specifically to the monoterpenoids and sesquiterpenoid subclasses. These results are consistent with previous studies identifying terpenoids as one of the major bioactive constituents in *S. officinalis* leaves.^48^

**Figure 3:**
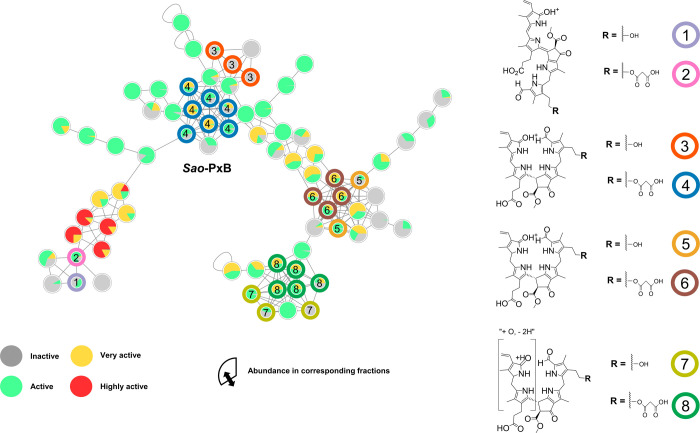
Informed *S. officinalis* network with incorporated bioactivity data and including tentatively assigned structures of phyllobilins (pie chart section size represents abundance in the corresponding fraction, and fill color corresponds to *in vitro* antioxidative activity). Nodes with colored borders were putatively annotated as PBs 1–8 (structure suggestions are depicted on the right). Nodes corresponding to *Sao*-PxB are highlighted in blue.

Closer examination of the PB cluster revealed that nodes annotated as *Ep*-PxB-6, based on exact matches with reference spectra, were primarily found in F9 and F10, which were classified as inactive in the FRAP assay (Figure 3). Interestingly, previous studies have reported notable antioxidative activity for this specific PB.^10,51^ Still, in a different study, a decrease in *in vitro* antioxidative activity with decreasing polarity was also observed.^9^ Nevertheless, additional PxB annotations generated through the GNPS analog search were detected in active and very active fractions (Figure 3 and Figure S8) also in higher abundance, based on ion intensity and peak area in the UV/vis chromatogram at 420 nm, indicating the presence of potentially bioactive PBs in senescent *S. officinalis* leaf extracts besides those confirmed by direct spectral matches.

We also evaluated the results from the intracellular ROS-scavenging assay, applying a similar color-coding strategy to visualize activity within the network. This revealed a distinct cluster of highly active compounds, which were annotated using CANOPUS and NPClassifier (Figures S10 and S11 and Table S3). Many of these compounds were classified as diterpenoids, aligning with bioactive constituents in *S. officinalis*.^48^ Interestingly, the three *Ep*-PxB-6 nodes based on exact spectral matches now appeared in active fractions, highlighting the importance of assessing the antioxidative potential with complementary assays. Additional analog-based PB annotations were also found in bioactive fractions.

In order to guide the isolation and structure elucidation of bioactive PBs, we subsequently aimed to postulate putative structures, focusing on the most bioactive nodes within the PB cluster (Figure 3 and Table S5). Structure suggestions were based on an in-house PB library and were proposed when calculated MS^1^ masses and predicted MS^2^ fragment ions matched the experimental data within a mass defect tolerance of 5 ppm. Three nodes were annotated as *Ep*-PxB-6 based on GNPS reference spectra (Figure 3, #3). To postulate new structures, we propagated this structural information to neighboring nodes. As PBs mainly vary through substitutions at rings A and D, nodes with similar MS^2^ spectra but different masses likely represent derivatives with different residues at these positions. Notably, several bioactive nodes with a neutral mass of 728.3 Da (Figure 3, #4) were identified near the *Ep*-PxB-6 subcluster (Figure 3, #3). This mass difference of +86 Da compared to *Ep*-PxB-6 likely indicates the addition of a malonyl ester group. A recent study reported a PxB with the same mass in senescent leaves of *O. basilicum*, another Lamiaceae species.^52^ Based on MS^2^ fragmentation pattern comparison, this compound, named *Sao*-PxB (from *Salvia officinalis*), was proposed to be identical. The close structural relationship between *Sao*-PxB and *Ep*-PxB-6 is additionally supported by a direct comparison of their MS^2^ spectra (Figure 4). Key fragments corresponding to the loss of methanol, methanol + CO_2_, and ring C + D are consistently shifted by +86 Da in *Sao*-PxB, aligning with the proposed malonyl substitution. However, the fragment corresponding to the loss of ring A remains identical in both spectra, underscoring the structural conservation of that region and supporting their close chemical similarity. As discussed earlier, *Ep*-PxB-6 may be formed as an in-source fragment of *Sao*-PxB during electrospray ionization via ester bond cleavage. In order to verify whether *Ep*-PxB-6 is genuinely present in *S. officinalis*, the methanolic extract was analyzed using (U)­HPLC-VWD-ESI-HRMS^2^ with adjusted parameters (Figure S12). Peaks corresponding to *Ep*-PxB-6 and *Sao*-PxB eluted at distinct retention times, which confirmed that *Ep*-PxB-6 is not merely an in-source fragment of *Sao*-PxB but occurs naturally in senescent *S. officinalis* leaves. However, the possibility remains that *Ep*-PxB-6 is formed through nonenzymatic ester hydrolysis of the malonyl group during extraction, fractionation, or analysis. Since phyllobilins primarily differ in their substitution patterns at this ester position, such cleavage yielding *Ep*-PxB-6 can be advantageous as it enables the annotation and identification of phyllobilin clusters using the newly established FBMN workflow, only by utilizing the reference MS^2^ spectrum of *Ep*-PxB-6 in the GNPS spectral library. In order to further characterize the PB profile of *S. officinalis*, we investigated additional nodes likely representing structurally related compounds. Nodes with mass differences of +2 Da, compared to their PxB counterparts, were identified as potential PleBs, the reduced form of PxBs, including *Ep*-PleB-6 (Figure 3, #5) and *Sao*-PleB (Figure 3, #6). Previous studies have reported that *Ep-*PleB-6 exhibits lower *in vitro* antioxidative potential than *Ep*-PxB-6.^10^ However, both *Ep*-PleB-6 and *Sao*-PleB were found in fractions classified as active or very active in the FRAP assay. This is probably the result of the complex nature of the fractions used for biological testing as these fractions contain multiple coeluting metabolites that affect antioxidative potential in addition to PBs. We also detected potential PrBs with mass differences of −2 Da, compared to their PxB counterparts, (Figure 3, #1 and #2), previously only observed in trace amounts.^44^ These PrBs mainly appeared in inactive fractions. Although their intrinsic antioxidative potential has not been studied, their largely unsaturated structures suggest limited redox activity, which is consistent with their distribution within inactive fractions. Another cluster of putative PBs with mass differences of +16 Da, corresponding to an additional carbonyl group, could also be observed (Figure 3, #7 and #8). No antioxidative properties have been reported for this PB subclass so far. Interestingly, compounds bearing a malonyl side chain were primarily found in fractions with higher FRAP activity, whereas those containing a hydroxyl group appeared in less active fractions. This trend reversed in the cellular ROS assay, mirroring the behavior observed for the PxBs (Figure S11). Although direct structure–activity relationship (SAR) conclusions remain difficult, this study provides a foundation for future detailed SAR investigations.

**Figure 4:**
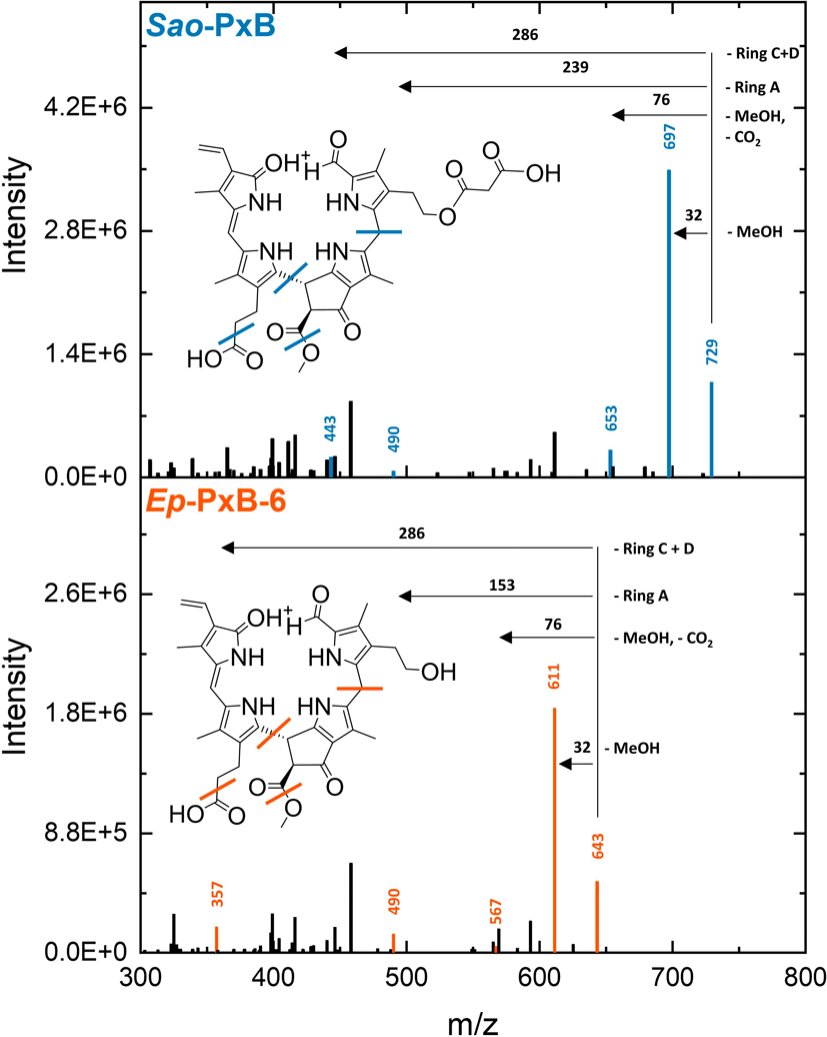
MS^2^ spectra of *Sao*-PxB ([M + H]^+^, *m*/*z* 729) and *Ep*-PxB-6 ([M + H]+, *m*/*z* 643) are presented with characteristic fragmentation patterns highlighted. To enhance readability, *m*/*z* values have been rounded.

Comparing the PB clusters between the two species reveals that, although the two species differ considerably in their overall metabolite composition, both yielded well-resolved PB clusters using the same preprocessing, UV/vis highlighting, and FBMN settings. In both extracts, we observed comparable PB classes (e.g., PleBs and PxBs) and similar MS^2^ fragmentation patterns characteristic for the tetrapyrrolic core. Differences between the species mainly concern the relative abundance and distribution of individual PBs rather than the ability of the workflow to detect or cluster them. These observations support that the workflow performs robustly across chemically distinct matrices, indicating its broader utility for PB analysis in diverse plant systems.

### Targeted Isolation and Structure Elucidation of *Sao*-PxB

*Sao*-PxB was chosen for isolation, since it was found as the most abundant PB, based on ion intensity and peak area in the UV/vis chromatogram recorded at 420 nm. Moreover, isolation of a PxB structure was favored, as studies showed that PxBs tend to be more active compared to their PleB counterparts regarding antioxidative activity, as well as antiproliferative properties.^10,12^ In addition, the other PBs were present only in trace amounts.

Two grams of the previously prepared dried methanol extract was used and subjected to preparative HPLC, which yielded 1.3 mg of *Sao*-PxB. To confirm the structure of *Sao*-PxB, 1D- and 2D-NMR experiments were performed (Figures S13 and S14). As described above, a PxB with the identical mass of 728 Da (*Ob*-YCC-45) has already been isolated from basil (*Ocimum basilicum*) leaves and structurally characterized.^52^ As a type I PB, *Ob*-YCC-45 carries a formyl group as well as a malonic ester and a vinyl group as residues at rings A and D. ^1^H NMR, ^13^C NMR, and H–H and H–C correlation experiments were conducted to confirm that *Sao*-PxB shares the identical structure as *Ob*-YCC-45, as already suggested by FBMN analysis and interpretation of the MS^2^ fragmentation pattern. The ^1^H NMR showed five singlets with an integral of 3, which could be assigned to four methyl groups directly attached to the pyrrol rings A, B, C, and D (Figure S13A). One of these singlets showed a chemical shift of 3.66 ppm, which could be assigned to the methyl group of the methyl-ester at position C8^2^, for which no correlations in the COSY or NOESY spectra were observed. Another singlet at 9.46 ppm confirmed the presence of a formyl group, which also showed NOESY correlations with the methyl group at C2^1^, confirming that the formyl residue is located at ring A. The characteristic structural feature of a PxB, the double bond at the meso-C15 position, was found with a singlet with an integral of 1 at 6.06 ppm, typical for alkenes. Additionally, the NOESY correlations of the methyl groups at C13^1^ (ring C) and C17^1^ (ring D) confirm the presence of a PxB in contrast to a PleB. The COSY experiment also revealed a correlation between two protons with a similar chemical shift of 5.30/6.20 and 6.56 ppm, indicating a vinyl group at ring D. The presence of a malonyl ester modification in *Sao*-PxB was confirmed by HMBC experiments, which revealed correlations between protons and two carbons exhibiting chemical shifts characteristic of carbonyl groups. COSY and NOESY spectra further indicated that the protons resonating at 3.04 ppm show no significant correlations with other protons, supporting their isolated chemical environment. Additional evidence for the malonyl functionality was observed in the HMBC spectrum, where protons at C3^2^ and C3^4^ both correlate with the same carbonyl carbon C3^3^.

## Conclusions

In this study, we developed a novel workflow enabling the identification of PBs in complex and unprocessed plant extracts. A comparison of *E. purpurea* and *S. officinalis* demonstrates that the workflow yields well-defined PB clusters in both species despite their markedly different metabolite backgrounds. By enabling consistent detection, annotation, and comparison of PBs across diverse plant matrices, this workflow provides a solid foundation for future comprehensive analyses of PB diversity and for establishing detailed SARs. For the first time, PB profiles have been analyzed using FBMN, leading to the discovery of a previously unreported PB in senescent sage leaves. Isolation and structural elucidation of this compound, termed *Sao*-PxB, demonstrated both the feasibility of bioactivity-guided isolation and the potential of this approach to suggest structures of unknown PB structures within complex plant matrices. From now on, users of the GNPS-FBMN workflow can annotate several PBs in their extracts. This raises awareness of the widespread occurrence of chlorophyll metabolites and their biological significance, in particular as bioactive constituents of medicinal plants. The workflow presents a scalable strategy to uncover underexplored regions of the plant metabolome, particularly in senescent plants. Currently, seven PBs are listed in the GNPS public database, with more expected to be added in the near future. With the corresponding data also shared on LOTUS and the PhotochemCAD platform, the natural product research community now has access to a comprehensive PB data set—providing a valuable resource for the rapid and thorough identification of PBs in future studies.

## Supplementary Material



## Notes

Flash chromatograms from the fractionation of plant extracts; detailed fractionation method; UHPLC-HRMS^2^ analysis and data processing procedures; feature-based molecular network generation workflow; isolation protocol and full spectroscopic data for *Sao*-PxB; FRAP and ROS assay methods; structures of *Ep*-PxBs; feature-based molecular networks of *E. purpurea* and *S. officinalis* (including 420 nm filtered networks and analog-annotation views); TIMA and CANOPUS annotation tables for *Ep*-PxB nodes, phyllobilin clusters, and most active nodes identified in FRAP and ROS assays; GNPS-based spectral annotation results; activity-informed FBMN visualizations; chromatograms of *S. officinalis* extract, *Ep*-PxB-6, and *Sao*-PxB; structural-network analysis tables; and full NMR signal summaries and spectra (^1^H, ^1^H–COSY, ^1^H-NOESY, HMBC, and HSQC) for *Sao*-PxB (DOCX)
