# Supplementary material for: Informed Feature-Based Molecular Networking as a Complementary Approach to Identify Bioactive Phyllobilins in Senescent Plant Extracts
Source: ACS Omega. 2025 Dec 11;10(50):62312–21. doi: 10.1021/acsomega.5c10395 (PMC12750395; doi:10.1021/acsomega.5c10395)
Supplement: Supplementary file 1 [file ao5c10395_si_001.docx]

**Supporting Information**

**Informed feature-based molecular networking as a complementary approach to identify bioactive phyllobilins in senescent plant extracts**

Christian A. Elvert ^1,2^, Johanna K. S. Lückenbach ^1,2,3^, Simone Moser ^1,2^, Fabian Hammerle ^1,2^*, Cornelia A. Karg ^1,2^*

^1^ Center for Molecular Biosciences, University of Innsbruck, Innrain 80/82, A-6020 Innsbruck, Austria

^2^ Department of Pharmacognosy, Institute of Pharmacy, University of Innsbruck, Innrain 80/82, A-6020 Innsbruck, Austria

^3^ Institute of Medical Biochemistry, Medical University of Innsbruck, Innrain 80/82, A-6020 Innsbruck, Austria

* Shared correspondence

**Table of Contents**

Figure S1: Flash chromatograms from the fractionation of plant extracts ………………. S-3

Text S1: Fractionation method …………………………………………………………... S-4

Text S2: UHPLC-HRMS^2^ analysis and data processing ………………………………… S-4-6

Text S3: Feature-based molecular network generation ………………………………….. S-7-8

Text S4: *Sao*-PxB: isolation method ……………………………………………………... S-9

Text S5: *Sao*-PxB: spectroscopic data …………………………………………………… S-9-10

Text S6: FRAP assay method …………………………………………………………….. S-10-11

Text S7: ROS assay method ……………………………………………………………… S-11-12

Figure S2: Structures of *Ep*-PxBs………………………………………………………… S-12

Figure S3: *E. purpurea*: feature-based molecular network……………………………….. S-13

Figure S4: *E. purpurea*: feature-based molecular network 420 nm filter …………........... S-13

Table S1: *E. purpurea*: TIMA and CANOPUS annotations for the *Ep*-PxB nodes ……… S-14-15

Figure S5: *S. officinalis*: feature-based molecular network ……………………………..... S-15

Figure S6: *S. officinalis*: feature-based molecular network 420 nm filter ………………... S-16

Figure S7: *S. officinalis*: feature-based molecular network “analog annotation” ……….... S-16

Figure S8: *S. officinalis*: annotations based on the GNPS reference spectra ……………... S-17

Table S2: *S. officinalis*: TIMA and CANOPUS annotations phyllobilin cluster ………..... S-18-21

Figure S9: *S. officinalis*: FRAP assay results and informed FBMN ……………………… S-22

Table S3: *S. officinalis*: TIMA and CANOPUS annotations most active nodes (FRAP) … S-22-27

Figure S10: *S. officinalis*: ROS assay results and informed FBMN ……………………… S-27

Figure S11: *S. officinalis*: ROS assay results phyllobilin cluster and most active nodes … S-28

Table S4: *S. officinalis*: TIMA and CANOPUS annotations most active nodes (ROS) ….. S-28-30

Table S5: *S. officinalis*: Structural network analysis ……………………………………... S-31-32

Figure S12: *S.officinalis*: chromatograms *S. officinalis* extract, *Ep*-PxB-6, and *Sao*-PxB . S-33-34

Figure S13: *Sao*-PxB: summary NMR signals and correlations …………………………. S-35

Figure S14: *Sao*-PxB: ^1^H NMR, ^1^H-COESY, ^1^H-NOESY, HMBC, and HSQC spectra ... S-36

References ………………………………………………………………………………... S-37


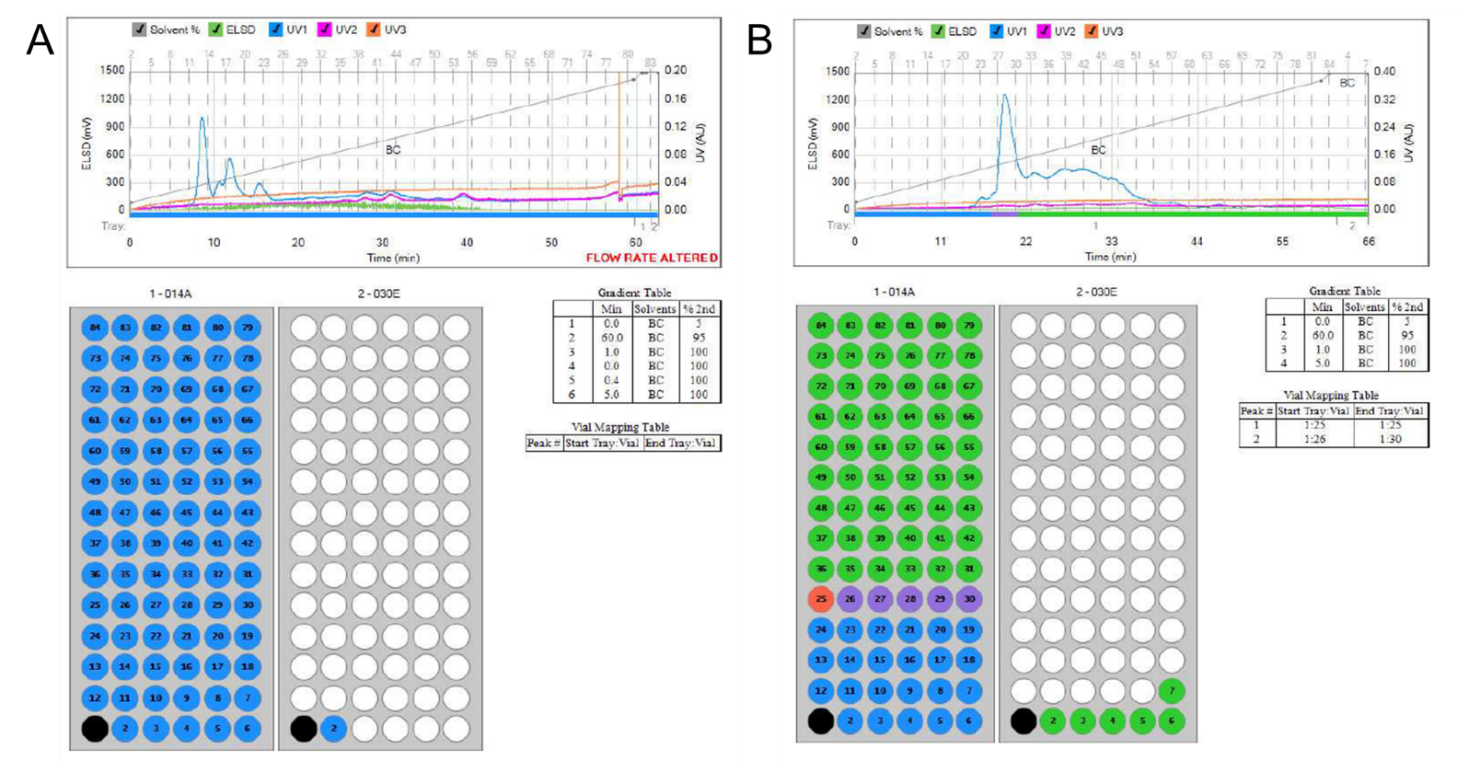


**Figure S1.** Chromatograms of *Echinacea purpurea* extract fractionation (A) and *Salvia officinalis* extract fractionation (B). Indicated in blue is the UV wavelength at 320 nm, magenta depicts the VIS signal at 420 nm and yellow shows the 500 nm VIS trace. The green line shows the ELSD signal. Numbered circles depict the collected vials during fractionation. Six combined vials yielded one fraction. The first two vials, as well as the last 7 vials were discarded. The flow rate had to be changed during the last 2 minutes of the separation, as an issue with the flow line occurred (A). Vials in red and purple were collected with different time slots, because of automated peak detection, which was turned off after vial 30. The fractions were combined based on the corresponding methanol percentage during elution to match the elution profile of the *E. purpurea* extract. Thus, the last 11 fractions were discarded as well (B).

**Text S1.** Fractionation method

Flash chromatography with UV/Vis and ELS Detection. Reveleris® X2 flash chromatography system with integrated UV/Vis- and ELS detector (Reveleris, Büchi, Flawil, Switzerland); Büchi FlashPure C18 cartridge (40 µm, irregular, 40g) (Büchi, Flawil, Switzerland); solvent system for the *E. purpurea* fractionation: A: 100 mM phosphate buffer in water at pH 7 (54 mM potassium phosphate dibasic (Merck, Darmstadt, Germany) and 46 mM potassium phosphate monobasic (Merck, Darmstadt, Germany), B: methanol; solvent system for the *S. officinalis* fractionation: B: 50 mM phosphate buffer in water at pH 7 (27 mM potassium phosphate dibasic and 23 mM potassium phosphate monobasic), C: methanol; flow rate: 20 mL/min; gradient: 0 – 60 min: 5% C to 95% C, 60 – 61 min 95% C to 100% C; 61– 66 min 100% C.

**Text S2.** UHPLC-HRMS^2^ analysis and data processing

The mass spectrometric parameters were as follows: heated-ESI source, static spray voltage (positive: 4500 V), sheath gas (N2): 30 arbitrary units, auxiliary gas (N2): 17 arbitrary units, sweep gas (N2): 0 arbitrary units. Temperature of the ion transfer tube and vaporizer was adjusted to 370 and 420 °C, respectively. MS data (range 100-1500 m/z) were recorded from 1 to 26 min with a resolution of 60 000 FWHM for MS^1^. The RF lens parameter was set to 70%. Data-dependent experiments were conducted with stepped collision energy mode and normalized collision energy type using HCD collision energies of 15, 30, and 45% at a resolution of 15 000 FWHM. The number of dependent scans was set to 3. The following selection of filters was employed: intensity threshold filter (1.0E5), dynamic exclusion (auto), isotope exclusion (assigned), charge state (perform dependent scans on singly charged precursors only), and apex filter (desired apex window: 75%). In addition, a specific exclusion list was created for the measurement using DMSO as a background extract with an IODA Mass Spec notebook.[1]

MS data preprocessing. Analysis results of *E. purpurea* and *S. officinalis* fractions were processed separately but with the same parameters: The data obtained in positive ionization mode were converted from the standard data format .raw (Thermo Scientific) to the open format .mzML using the MS Convert software included in the ProteoWizard package.[2] The converted files were processed with mzmine 4.3.0 (mzio GmbH, Bremen, Germany).[3] A batch file with following specified steps was created: The generated .mzML files were imported with the standard import function. For mass detection at the MS^1^ level, the noise level was set to 1.5E6. For MS^2^ detection, the noise level was set to 0.0E0. Parameters for the ADAP chromatogram builder module were set as follows: minimum consecutive scans, 5; minimum intensity for consecutive scans, 1.5E6; minimum absolute height, 1.5E6; scan to scan accuracy (*m/z*) of 0.0065 or 10.0 ppm. The Savitzky Golay algorithm was used for smoothing, with retention time smoothing set to 5. Chromatogram deconvolution was done with the local minimum feature resolver (MS/MS scan pairing, allowed; MS^1^ to MS^2^ precursor tolerance, 0.0100 *m/z* or 10.0 ppm; retention time filter, use feature edges; minimum relative feature height, 25.0%; minimum required signals, 1; dimension, retention time; chromatographic threshold, 88.3%; minimum search range RT, 0.150; minimum relative height, 0.0%; minimum absolute height, 1.5E6; min ratio of peak top/edge, 2.00; peak duration range, 0.00-1.51; minimum scans, 5). Isotopes were detected with the ^13^C isotope filter (m/z tolerance, 0.0032 m/z or 5.0 ppm; RT tolerance, 0.050 min; maximum charge, 1; representative isotope, most intense). Isotope signals for H, C, N, O, and S were searched using the isotopic peaks finder (*m/z* tolerance, 0.0032 *m/z* or 5.0 ppm; maximum charge of isotope *m/z*, 1; search in scans, single most intense). An aligned feature list was generated employing the Join aligner algorithm (*m/z* tolerance, 0.0065 *m/z* or 10.0 ppm; weight for m/z, 3; retention time tolerance, 0.07 min; weight for RT, 1). Gap filling was performed with the Peak finder (multithreaded) algorithm with the intensity tolerance, *m/z* tolerance (sample-to-sample), retention time tolerance, and minimum scans (data points) set to 20.0%, 0.0065 *m/z* or 10.0 ppm, 0.07 min, and 3, respectively. Duplicates were filtered using the Duplicate peak filter module (filter mode, new average), with the *m/z* tolerance set to 0.0016 *m/z* or 2.5 ppm and the RT tolerance set to 0.04 min. The feature list row filter was then applied keep only features with MS^2^ scan and to reset the feature number ID.

The resulting filtered list was subjected to ion identity molecular networking,[4] starting with the metaCorrelate module (RT tolerance, 0.06 min; minimum feature height, 0.0E0; intensity threshold for correlation, 1.5E6; feature shape correlation and feature height correlation, both allowed). Subsequently the ion identity networking algorithm was used (*m/z* tolerance, 0.0032 *m/z* or 5.0 ppm; check, all features; minimum height, 0.0E0; ion identity library: maximum charge, 2; maximum molecules/cluster, 2; adducts, [M+H-H20]^+^, [M+H]^+^, [M+Na]^+^, [M+K]^+^, [M-H+2Na]^+^, [M+2H]^2+^, [M-H+2Na]^+^; annotation refinement: delete small networks without major ion, yes; delete smaller networks, link threshold, 4; delete networks without monomer, yes). Feature annotations were obtained with the Local compound database search (database of PBs) as well as with the Lipid annotation module. Lastly, the aligned feature list was exported with the dedicated functions for molecular networking and for analysis with SIRIUS.

**Text S3.** Feature-based molecular network generation

The mass spectrometry data were first processed with mzmine 4.3.0 (see Text S2) and the results were exported to GNPS for FBMN analysis. The data were filtered by removing all MS/MS fragment ions within +/- 17 Da of the precursor *m/z*. MS/MS spectra were window filtered by choosing only the top 6 fragment ions in the +/- 50 Da window throughout the spectrum. The precursor ion mass tolerance was set to 0.02 Da and the MS/MS fragment ion tolerance to 0.02 Da. A molecular network was then created where edges were filtered to have a cosine score above 0.7 and more than 5 matched peaks. Further, edges between two nodes were kept in the network if and only if each of the nodes appeared in each other’s respective top 10 most similar nodes. Finally, the maximum size of a molecular family was set to 100, and the lowest scoring edges were removed from molecular families until the molecular family size was below this threshold. The spectra in the network were then searched against GNPS spectral libraries.[5, 6] The library spectra were filtered in the same manner as the input data. All matches kept between network spectra and library spectra were required to have a score above 0.6 and at least 4 matched peaks. The DEREPLICATOR was used to annotate MS/MS spectra.[7] Additional edges were provided by the user. The molecular networks were visualized using Cytoscape software.[8]

SIRIUS metabolite annotation. The corresponding .mgf file exported from mzmine 4.3.0 was processed with SIRIUS 6.1.1.[9] The parameters were set as follows: instrument, Orbitrap; MS^2^ mass accuracy, 10 ppm; fix formula for detected lipid, yes; fallback adducts: [M+H]^+^, [M+Na]^+^, [M+K]^+^; molecular formula generation, de novo + bottom up). Molecular formula ranking was improved using ZODIAC.[10] The prediction of fingerprints was carried out with CSI:FingerID and the prediction of chemical classes with CANOPUS.[11, 12] Only bio databases were searched for molecular formulas and structures.

Advanced annotation of secondary metabolites based on plant taxonomic information. Taxonomically informed metabolite annotations were generated with the R script “tima” (version 2.11.0),[13] following the instructions in the corresponding GitHub repository (taxonomicallyinformedannotation/tima: https://taxonomicallyinformedannotation.github.io/tima). The output data from the mzmine preprocessing were searched in an *in silico* database prepared from the combined records of the Dictionary of Natural Products and the LOTUS initiative. The re-ranking (i.e., forcing all features attributed to the taxon “Echinacea purpurea” or “Salvia officinalis”) was done with the publicly available taxonomical information stored in the LOTUS database (https://elifesciences.org/articles/70780). Predicted compound classes were assigned using either the ClassyFire or NPClassifier chemotaxonomies.[14, 15] ClassyFire classification was preferentially used for the identification of potential phyllobilin nodes, as it reflects the general molecular structure. In contrast, NPClassifier was favored for interpreting bioactivity-guided data, as its ontology is based on natural product biosynthetic origin and biofunctional relevance.

Filter variables for the network representation. Cytoscape-compatible information layers highlighting features in the network capable of absorbing light of either 320 or 420 nm were prepared using a dedicated workflow.[16, 17] The UV/Vis traces of each extract investigated were extracted and visualized as chromatograms with OriginPro 2020 (OriginLab Corporation, Northampton, Massachusetts, USA). Chromatograms were integrated to obtain peaks with start and end points. The threshold values were set at 5000 mAU for both 320 nm and 420 nm. Excel 365 was then used to determine, for each feature in the combined feature list, in which extract that specific feature was present with the largest peak area. The retention times of the UV/Vis peaks were then matched with those of the features (MS peaks). The dead volume between DAD and MS detector was negligible. If the feature could be assigned to a peak, the value was set to 1. If it could not be assigned, the value of this variable was set to 0. The results were exported as .csv file. For phyllobilin cluster identification, an in-house phyllobilin MS^1^ database was used, which included the SMILES and exact mass of [M+H]^+^ ions. All these data were collected from literature.

**Text S4.** *Sao*-PxB: isolation method

Preparative HPLC-DAD for compound isolation. Gilson PLC 2250 preparative HPLC with integrated DAD detector (Gilson, Berlin, Germany); Phenomenex Luna C18(2) column (100 Å, 5 µm, 21.2 x 250 mm) (Phenomenex, Aschaffenburg, Germany) protected by a Phenomenex SecurityGuard C18 pre column (Phenomenex, Aschaffenburg, Germany); solvent system: A: 10 mM ammonium formiate + 0.1% (V/V) formic acid in water, mobile phase B: acetonitrile; flow rate: 20 mL/min; gradient: 0 – 5 min 20% B, 5 – 8 min 20% B to 40% B, 8 – 28 min 40% B to 90% B, 28 – 35 min 90% B to 100% B 35 – 40 min 100% B. Data was processed with Gilson Glider CPC software. To desalt the fraction, it was applied once again to a solid-phase extraction cartridge, washed with water and eluted with methanol. The fraction was dried under vacuum and freeze dried to obtain 1.3 mg *Sao*-PxB.

**Text S5.** *Sao*-PxB: spectroscopic data

UV-Vis Spectrophotometer. ThermoFisher Scientific NanoDrop One (Thermo Scientific, Waltham, Massachusetts, USA); the molar extinxtion coefficient of log ε (426 nm) = 4.51 was used to determine the concentration of *Sao*-PxB.[18] Structure elucidation via NMR. Experiments were conducted on an Avance II+ 600 MHz spectrometer from Bruker (Bruker, Billerica, Massachusetts, USA) with a liquid nitrogen cooled TCI Prodigy probe using d6-DMSO + 0.03% TMS (Eurisotop, Saarbrücken, Germany) as solvent. MestReNova 14.3.3 (Mestrelab Research, Barcelona, Spain) was used for data processing.

^1^H NMR (600 MHz, d6-DMSO): 1.89 (s, H3C7^1^); 2.05 (s, H3C13^1^); 2.15 (s, H3C17^1^); 2.18 (s, H3C2^1^); 2.21 (m, HAC12^2^); 2.38 (m, HBC12^2^); 2.46 (m, H2C12^1^); 2.52 (m, H2C3^1^); 3.04 (dd, H2 C3^4^); 3.66 (s, H3C8^4^); 3.81 (m, HAC3^2^), 3.85 (m, HC8^2^); 3.93 (m, HBC3^2^); 3.95 (s, H2C5); 4.88 (d, HC10); 5.30 (dd, HAC18^2^); 6.06 (s, HC15); 6.20 (dd, HBC18^2^); 6.56 (m, HC18^1^); 9.46 (s, HC20); 10.16 (s, HN24)

^13^C (600 MHz, d6-DMSO, signal assignment based on HSQC and HMBC spectra): 8.8 (C2^1^); 9.0 (C7^1^); 9.3 (C13^1^); 9.5 (C17^1^); 19.3 (C12^1^); 22.6 (C3^1^); 23.2 (C5); 35.9 (C12^2^); 44.7 (C3^4^); 52.2 (C8^4^); 64.1 (C3^2^); 66.2 (C8^2^); 101.1 (C15); 110.1 (C7); 117.5 (C18^2^); 118.3 (C3); 121.3 (C12); 122.9 (C16); 123.2 (C18); 123.6 (C13); 124.1 (C14); 124.6 (C8); 127.2 (C18^1^); 127.6 (C1); 130.8 (C11); 131.0 (C2); 133.1 (C6); 136.3 (C4); 142.3 (C17); 156.7 (C9); 168.3 (C3^3^); 169.2 (C3^5^); 170.1 (C8^3^); 170.8 (C19); 174.7 (C12^3^); 176.5 (C20); 187.9 (C8^1^)

UHPLC-HR-ESI-MS. retention time 19.18 min, *m/z* calculated (C38H41N4O11) = 729.2766 [M+H]^+^; *m/z* found = 729.2753 [M+H]^+^ (Δ = -1.78 ppm), m/z found = 1457.5438 [2M+H]^+^

UV-Vis *Sao*-PxB (in MeOH) λ_max_, nm (relative ε) 211 (1.00), 244 (0.47), 313 (0.47), 429 (0.38)

**Text S6.** FRAP assay method

The different fractions and the extracts were diluted to a concentration of 0.1 mg/mL with 75% ethanol. Then, the fractions, as well as different concentrations of Trolox were mixed with the FRAP reagent (10 volumes of 300 mM acetate buffer pH 3.5, 1 volume of 10 mM 2,4,6-Tri-(2-pyridyl)-s-triazin (TPTZ) in 40 mM HCl and 1 volume of 20 mM iron (III) chloride) and incubated at 37 °C for 5 minutes. The absorbance of the resulting complex was measured at 593 nm on a TECAN Spark plate reader. Increasing concentrations of Trolox (0.4 µM – 650 µM) were used to generate a calibration curve. The antioxidative potential was expressed as Trolox equivalents in µMol of Trolox per mg/mL of extract. The activity of each fraction was ranked and color coded as follows: grey (inactive: Trolox equivalents < 1200 µMol/mg/mL), green (active: Trolox equivalents between 1200 and 2000 µMol/mg/mL), orange (very active: Trolox equivalents between 2000 and 2800 µMol/mg/mL), and red (highly active: Trolox equivalents > 2800 µMol/mg/mL). The experiment was performed in three independent experimental runs, each conducted with technical triplicates for all tested fractions. Results are presented as the mean of the three experiments, with standard deviations shown as error bars.

**Text S7.** ROS assay method

The antioxidative activity in cells was tested by using a protocol of Karg et. al with minor adaptations.[19] First, 2*10^5^ HaCaT cells were seeded in a 96 well format (RPMI-1460 (Merck, Darmstadt, Germany) supplemented with 10% FBS (Thermo Scientific, Waltham, Massachusetts, USA)). After 48 hours the medium was discarded and 25 µM of 2’,7’-dichlorofluorescein diacetate (DCFH-DA) dissolved in Hank’s buffered salt solution (HBSS, Merck, Darmstadt, Germany) along with 100 µg/mL (final concentration per well) of the fractions or extracts were added to the cells. After an hour of incubation at 37°C, cells were washed with HBSS and afterwards 2,2’-(Diazene-1,2-diyl)bis(2-methylpropanimidamide) dihydrochloride (AAPH) (Fluorochem, Hadfield, United Kingdom) dissolved in HBSS for a final concentration of 600 µM per well was added. The same amount of HBSS without AAPH was added to the same number of wells and served as a control. The fluorescence intensity (excitation at: 485 nm; emission at: 535 nm) of the oxidized DCFH-DA was measured after 45 minutes of incubation at 37 °C on a TECAN Spark plate reader. Potential antioxidative compounds scavenge ROS and reduce the formation of oxidized DCFH-DA. To assess the influence of potential toxic effects of the extracts and fractions on HaCaT cells a CellTiter-Blue® (CTB) (Promega, Walldorf, Austria) cell viability assay was performed afterwards. Therefore, the HBSS was removed and 50 µL of medium as well as 5 µL of the CTB reagent was added to each well. Cell viability was assessed after 2 and 20 hours of incubation at 37 °C by measuring the fluorescence emission at 560 nm on a plate reader. Results are expressed as relative DCF-DA fluorescence intensity in percent compared to a vehicle control, cells which were treated with DMSO, and set to 1. The assay was conducted using three biological replicates, each measured with technical triplicates. Results are reported as the mean of the biological replicates, with standard deviations shown as error bars.


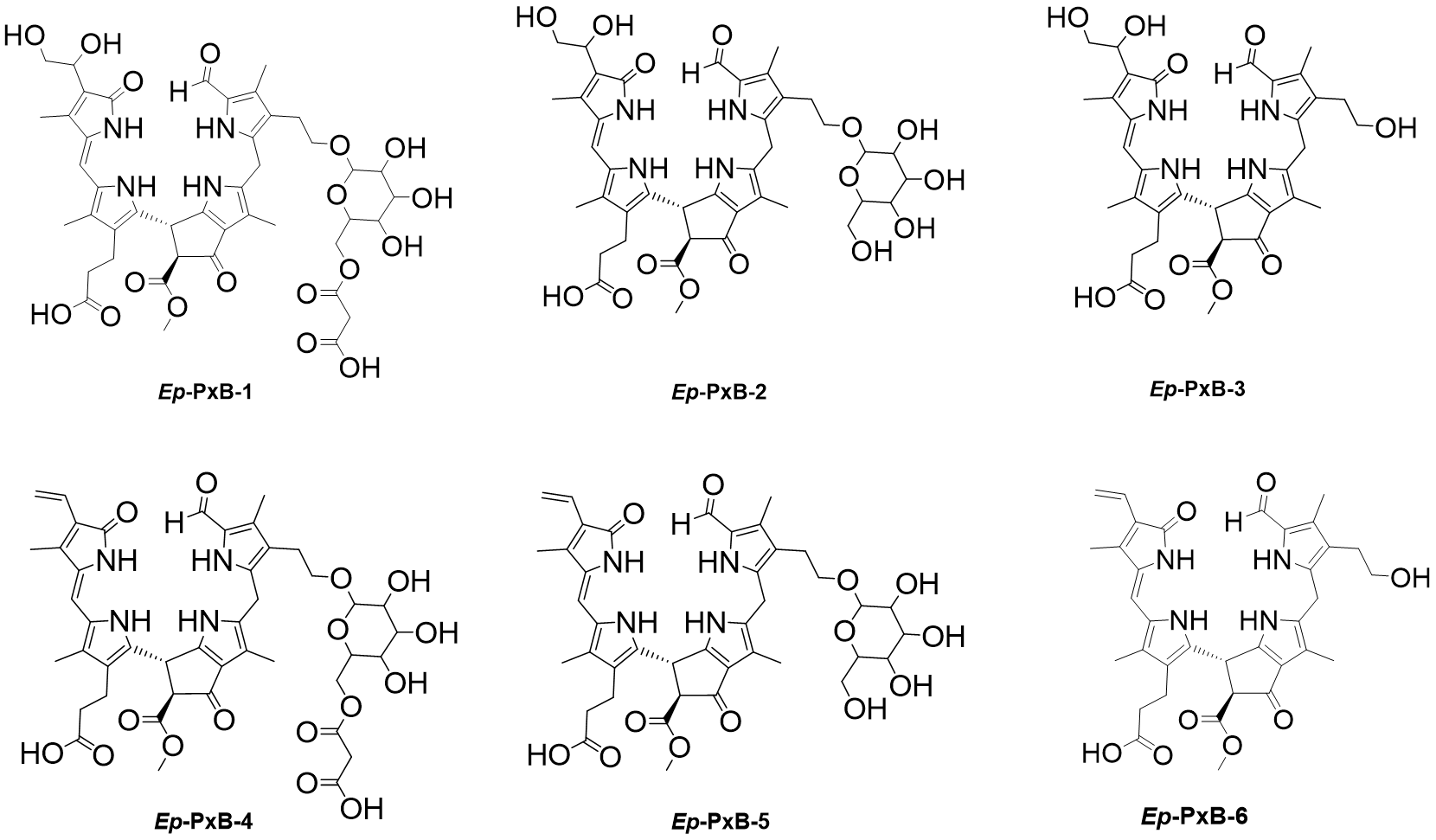


**Figure S2.** Structures of *Ep*-PxBs


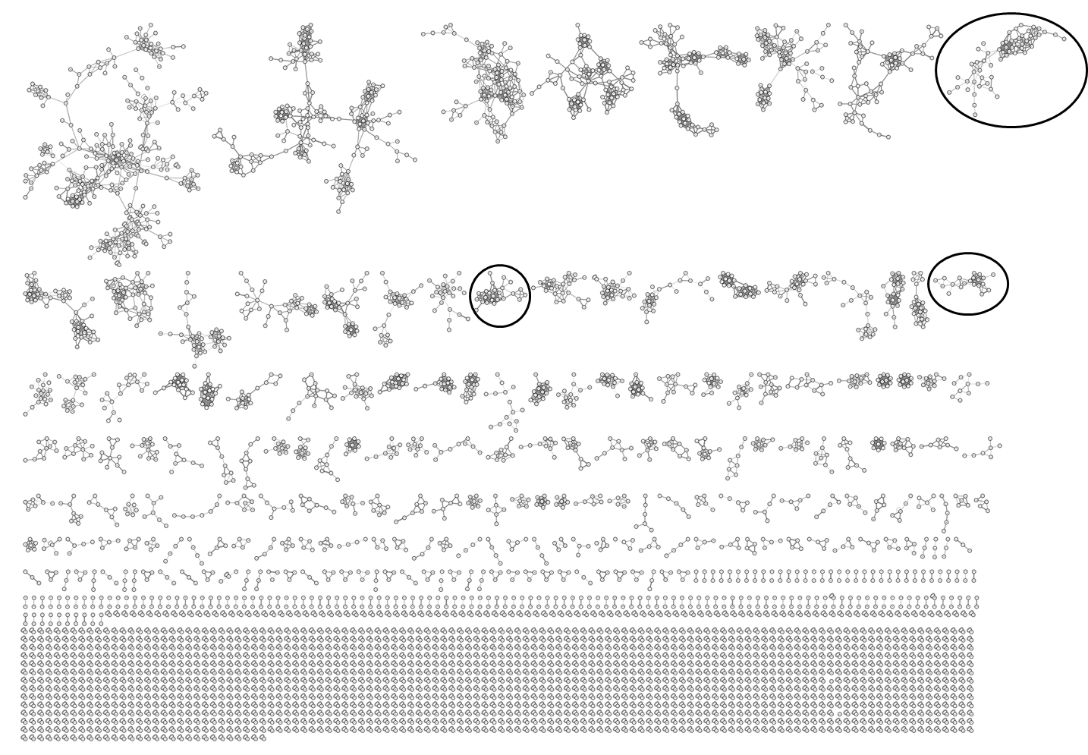


**Figure S3.** Feature-based molecular network of the 13 *E. purpurea* fractions, consisting of 4378 individual nodes and 7501 edges. Highlighted are phyllobilin clusters, containing *Ep*-PxBs.


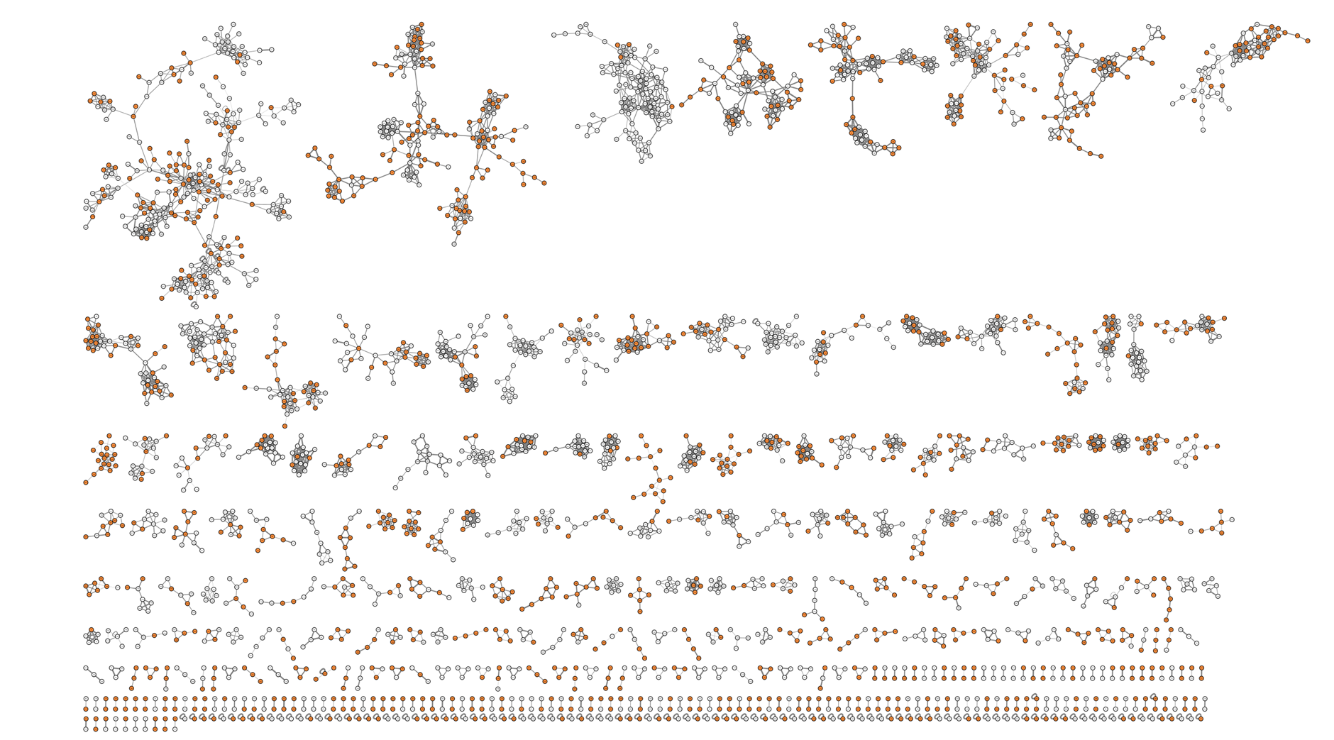


**Figure S4.** Feature-based molecular network of *E. purpurea* (only clusters visualized), nodes in orange exhibit a 420 nm absorption signal above a specific threshold.

**Table S1.** Potential compound class annotations using TIMA and CANOPUS, classified with the ClassyFire class categories for each *Ep*-PxB node from the generated feature-based molecular network of *E. purpurea*.

| Node-ID | Precursor mass [M+H]^+^ | In-house library annotation | Retention time [min] | Absorbance at 420 nm | TIMA: ClassyFire Class | CANOPUS: ClassyFire Class |
| --- | --- | --- | --- | --- | --- | --- |
| 4547 | 925.3335 | *Ep*-PxB-1 | 5.68 | yes | Not classified | Phenols |
| 4548 | 925.3336 | *Ep*-PxB-1 | 5.24 | yes | Fatty acyls | Tetrapyrroles and derivatives (Top 3 hit) |
| 4549 | 925.3337 | *Ep*-PxB-1 | 4.98 | yes | Not classified | Prenol Lipids |
| 4424 | 839.3326 | *Ep*-PxB-2 | 5.20 | yes | Fatty acyls | Carboxylic acids and derivatives |
| 4425 | 839.3328 | *Ep*-PxB-2 | 4.63 | yes | Fatty acyls | Carboxylic acids and derivatives |
| 3926 | 677.2807 | *Ep*-PxB-3 | 6.33 | no | Prenol lipids | Carboxylic acids and derivatives |
| 4510 | 891.3271 | *Ep*-PxB-4 | 16.19 | yes | Benzodioxanes | Tetrapyrroles and derivatives (Top 2 hit) |
| 4511 | 891.3273 | *Ep*-PxB-4 | 15.44 | yes | Fatty acyls | Carboxylic acid and derivatives |
| 4512 | 891.3278 | *Ep*-PxB-4 | 10.99 | no | Not classified | Tetrapyrroles and derivatives |
| 4332 | 805.3274 | *Ep*-PxB-5 | 9.68 | yes | Fatty acyls | Tetrapyrroles and derivatives (Top 4 hit) |
| 4333 | 805.3275 | *Ep*-PxB-5 | 15.71 | yes | Fatty acyls | Tetrapyrroles and derivatives (10 ppm) |
| 4334 | 805.3276 | *Ep*-PxB-5 | 15.6 | yes | Fatty acyls | Tetrapyrroles and derivatives |
| 4335 | 805.3277 | *Ep*-PxB-5 | 12.38 | no | Fatty acyls | Tetrapyrroles and derivatives (Top 4 hit) |
| 4336 | 805.3277 | *Ep*-PxB-5 | 10.82 | no | Fatty acyls | Tetrapyrroles and derivatives |
| 4337 | 805.3277 | *Ep*-PxB-5 | 6.89 | no | Fatty acyls | Tetrapyrroles and derivatives |
| 4338 | 805.3278 | *Ep*-PxB-5 | 13.71 | no | Fatty acyls | Tetrapyrroles and derivatives (Top 2 Hit, 10 ppm) |
| 4339 | 805.3278 | *Ep*-PxB-5 | 6.49 | no | Fatty acyls | Tetrapyrroles and derivatives |
| 4340 | 805.3279 | *Ep*-PxB-5 | 12.95 | no | Fatty acyls | Tetrapyrroles and derivatives (Top 2 Hit) |
| 3740 | 643.2748 | *Ep*-PxB-6 | 13.08 | no | Carboxylic acids and derivatives | Tetrapyrroles and derivatives |
| 3741 | 643.2750 | *Ep*-PxB-6 | 12.18 | no | Carboxylic acids and derivatives | Tetrapyrroles and derivatives |
| 3742 | 643.2751 | *Ep*-PxB-6 | 15.18 | yes | Carboxylic acids and derivatives | Tetrapyrroles and derivatives |
| 3743 | 643.2751 | *Ep*-PxB-6 | 15.92 | yes | Carboxylic acids and derivatives | Tetrapyrroles and derivatives |
| 3744 | 643.2753 | *Ep*-PxB-6 | 14.26 | yes | Prenol lipids | Tetrapyrroles and derivatives |
| 3745 | 643.2753 | *Ep*-PxB-6 | 16.90 | yes | Carboxylic acids and derivatives | Tetrapyrroles and derivatives |
| 3746 | 643.2753 | *Ep*-PxB-6 | 19.01 | yes | Carboxylic acids and derivatives | Tetrapyrroles and derivatives |
| 3747 | 643.2754 | *Ep*-PxB-6 | 19.08 | yes | Carboxylic acids and derivatives | Tetrapyrroles and derivatives |
| 3748 | 643.2754 | *Ep*-PxB-6 | 14.48 | no | Carboxylic acids and derivatives | Tetrapyrroles and derivatives |
| 3750 | 643.2755 | *Ep*-PxB-6 | 17.21 | yes | Carboxylic acids and derivatives | Tetrapyrroles and derivatives |


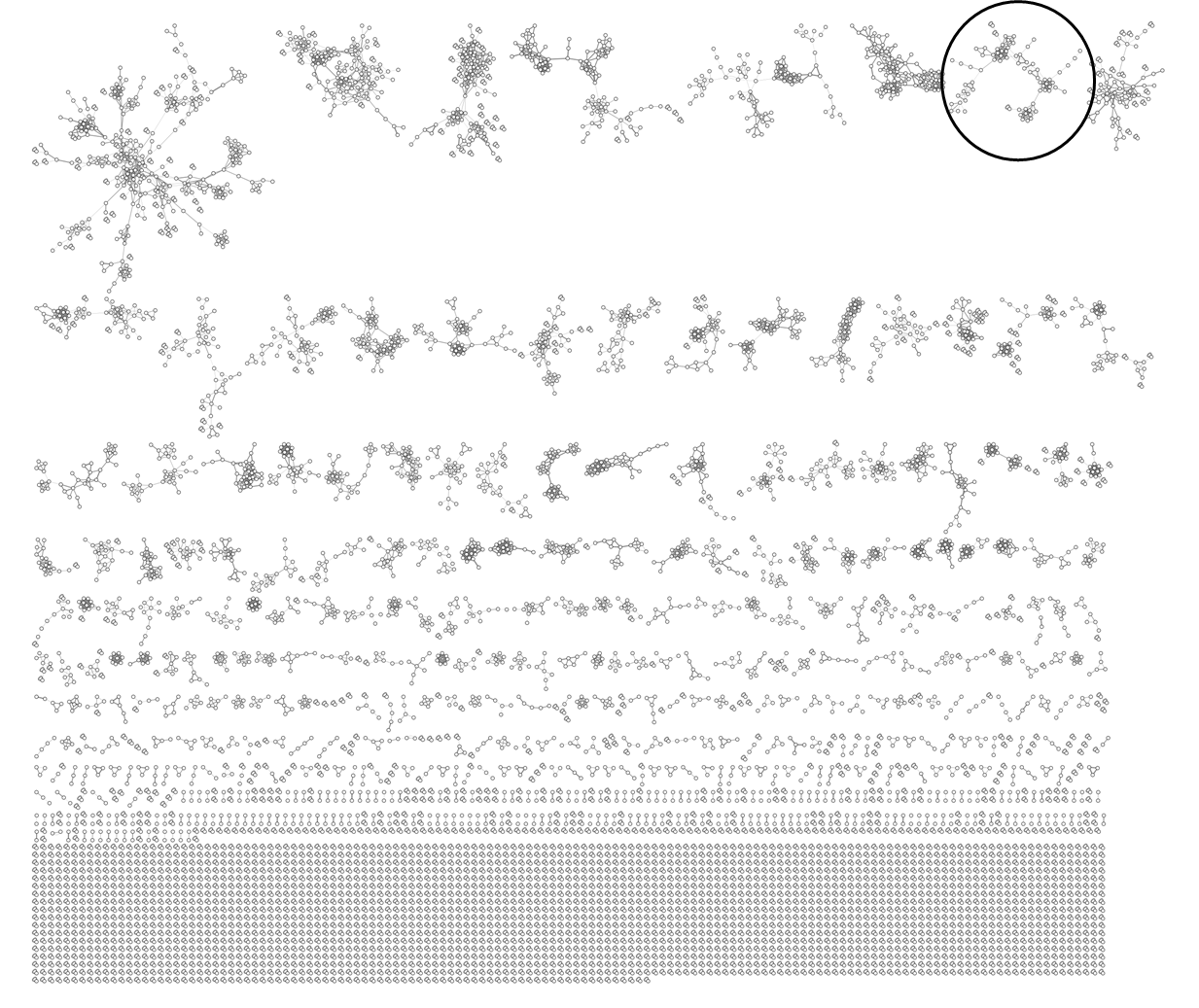


**Figure S5.** Feature-based molecular network of the 13 *S. officinalis* fractions, consisting of 6640 individual nodes and 10832 edges. Highlighted is the phyllobilin cluster.


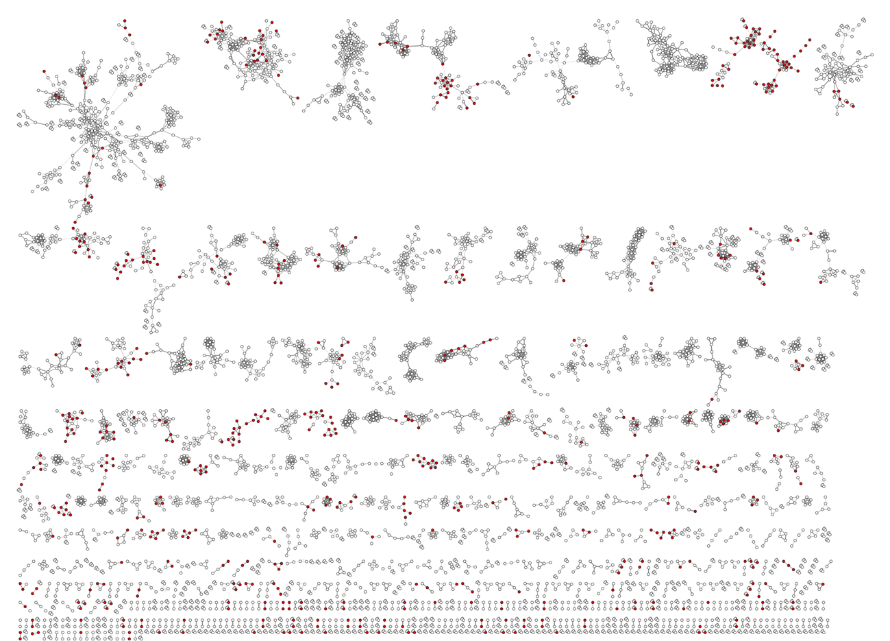


**Figure S6.** Feature-based molecular network of *S. officinalis* (only clusters visualized), nodes in red exhibit a 420 nm absorption signal above a specific threshold.


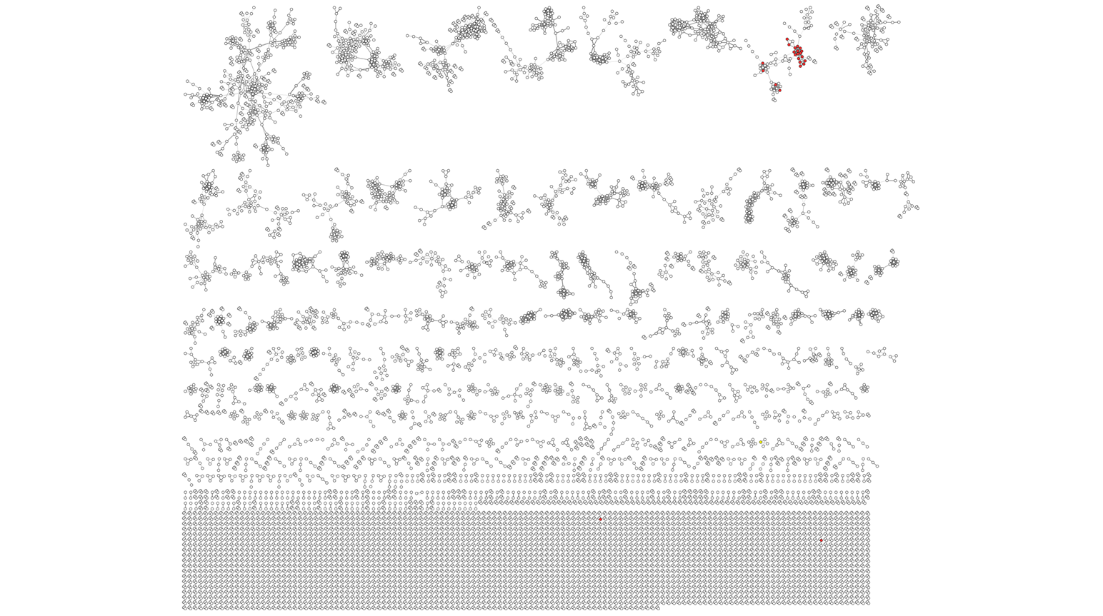


**Figure S7.** Feature-based molecular network of *S. officinalis*, nodes in red were annotated based on the “analog annotation” strategy within the GNPS workflow.


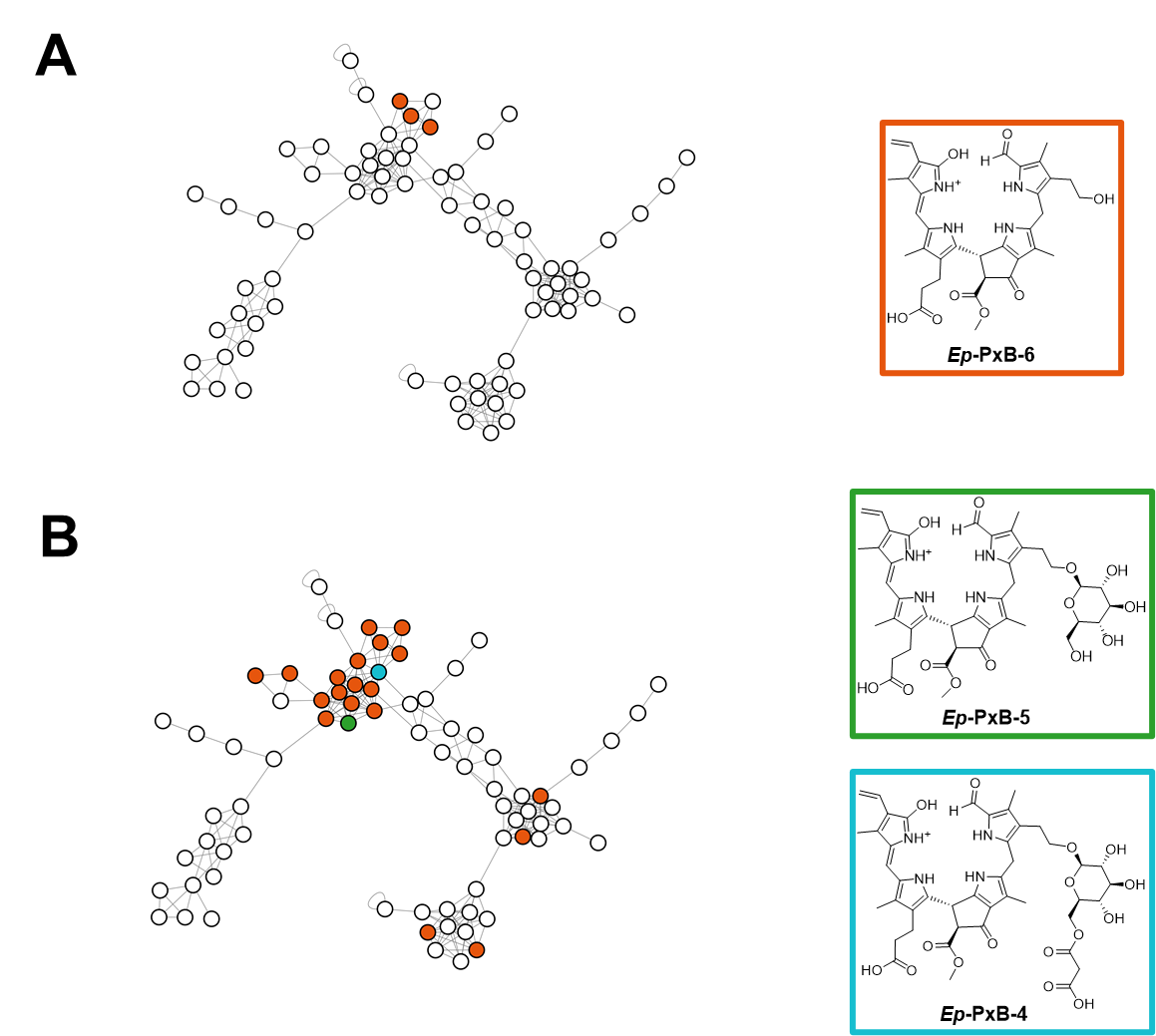


**Figure S8.** A) Annotations based on the phyllobilin reference spectra led to a direct match with 3 nodes of *Ep*-PxB-6 (orange). B) Annotations based on the GNPS analog search tool resulted in additional annotations of *Ep*-PxB-6 (orange), but also for *Ep*-PxB-5 (green) and *Ep*-PxB-4 (blue).

**Table S2.** Depicted are potential compound class annotations using TIMA and CANOPUS, classified with the ClassyFire class categories for each node from the phyllobilin cluster of the generated feature-based molecular network of *S. officinalis*.

| Feature-ID | Precursor mass [M+H]^+^ | Retention time [min] | Absorbance at 420 nm | TIMA: ClassyFire Class | CANOPUS: ClassyFire Class |
| --- | --- | --- | --- | --- | --- |
| 6554 | 1013.4527 | 20.19 | no | Not classified | Carboxylic acids and derivatives |
| 6493 | 967.4471 | 20.52 | no | Steroids and steroid derivatives | Carboxylic acids and derivatives |
| 6494 | 967.4475 | 20.27 | no | Prenol lipids | Carboxylic acids and derivatives |
| 5293 | 683.2699 | 19.59 | yes | Prenol lipids | Tetrapyrroles and derivatives |
| 4869 | 624.2183 | 8.11 | no | Peptidomimetics | Benzene and substituted derivatives |
| 4868 | 624.2182 | 12.93 | yes | Peptidomimetics | Tetrapyrroles and derivatives |
| 4867 | 624.2182 | 13.81 | yes | Peptidomimetics | Tetrapyrroles and derivatives |
| 4735 | 610.2025 | 6.62 | no | Macrolides and analogues | Tetrapyrroles and derivatives |
| 4736 | 610.2026 | 5.12 | no | Not classified | Tetrapyrroles and derivatives |
| 5013 | 642.2288 | 6.00 | no | Not classified | Carboxylic acids and derivatives |
| 5012 | 642.2286 | 6.62 | no | Not classified | Carboxylic acids and derivatives |
| 5573 | 727.2597 | 19.56 | yes | Stilbenes | Carboxylic acids and derivatives |
| 5801 | 759.2857 | 19.32 | yes | Fatty Acyls | Naphthalenes |
| 5367 | 695.2704 | 20.04 | yes | Carboxylic acids and derivatives | Tetrapyrroles and derivatives |
| 5005 | 641.2606 | 19.58 | yes | Prenol lipids | Tetrapyrroles and derivatives |
| 5793 | 757.2702 | 19.53 | yes | Flavonoids | Naphthopyrans |
| 5586 | 729.2746 | 19.60 | yes | Lignan glycosides | Tetrapyrroles and derivatives |
| 5689 | 743.2914 | 19.52 | yes | 2-arylbenzofuran flavonoids | Tetrapyrroles and derivatives |
| 5587 | 729.2753 | 19.18 | yes | Lignan glycosides | Naphthopyrans |
| 5592 | 729.2757 | 18.04 | yes | Lignan glycosides | Tetrapyrroles and derivatives |
| 5589 | 729.2754 | 19.06 | yes | Lignan glycosides | Tetrapyrroles and derivatives |
| 5590 | 729.2754 | 14.98 | yes | Lignan glycosides | Tetrapyrroles and derivatives |
| 5591 | 729.2756 | 14.59 | no | Lignan glycosides | Tetrapyrroles and derivatives |
| 5588 | 729.2753 | 14.90 | no | Lignan glycosides | Tetrapyrroles and derivatives |
| 5380 | 697.2857 | 19.62 | yes | Lignan glycosides | Tetrapyrroles and derivatives |
| 5379 | 697.2491 | 19.19 | yes | Lignan glycosides | Tetrapyrroles and derivatives |
| 5378 | 697.249 | 19.10 | yes | Lignan glycosides | Tetrapyrroles and derivatives |
| 5097 | 653.2598 | 19.51 | yes | Organooxygen compounds | Tetrapyrroles and derivatives |
| 5320 | 685.2856 | 19.50 | yes | Prenol lipids | Tetrapyrroles and derivatives |
| 6184 | 835.3903 | 20.10 | no | Prenol lipids | Carboxylic acids and derivatives |
| 5030 | 643.2754 | 17.14 | yes | Carboxylic acids and derivatives | Tetrapyrroles and derivatives |
| 5029 | 643.2753 | 19.01 | yes | Carboxylic acids and derivatives | Tetrapyrroles and derivatives |
| 5027 | 643.2751 | 14.22 | yes | Carboxylic acids and derivatives | Tetrapyrroles and derivatives |
| 5004 | 641.2598 | 16.98 | yes | Prenol lipids | Tetrapyrroles and derivatives |
| 6629 | 1391.5457 | 19.53 | yes | Not classified | Organooxygen compounds |
| 6621 | 1369.5638 | 19.53 | yes | Not classified | Tetrapyrroles and derivatives |
| 6258 | 857.3716 | 20.10 | no | Prenol lipids | Oxanes |
| 5753 | 751.2568 | 19.08 | yes | Organooxygen compounds | Carboxylic acids and derivatives |
| 5752 | 751.2568 | 19.21 | yes | Organooxygen compounds | Not classified |
| 5754 | 751.2571 | 19.76 | yes | Organooxygen compounds | Organooxygen compounds |
| 6638 | 1457.5445 | 19.20 | yes | Lignan glycosides | Not applicable |
| 6640 | 1479.5256 | 19.20 | yes | Not classified | Not applicable |
| 5760 | 753.2724 | 16.45 | no | Prenol lipids | Prenol lipids |
| 5181 | 667.273 | 15.88 | no | Prenol lipids | Tetrapyrroles and derivatives |
| 5182 | 667.273 | 15.80 | yes | Prenol lipids | Carboxylic acids and derivatives |
| 5762 | 753.2727 | 15.21 | yes | Prenol lipids | Not classified |
| 5761 | 753.2725 | 16.56 | yes | Prenol lipids | Not classified |
| 5601 | 731.2909 | 16.48 | no | Carboxylic acids and derivatives | Naphthopyrans |
| 5046 | 645.2911 | 15.85 | yes | Steroids and steroid derivatives | Tetrapyrroles and derivatives |
| 5709 | 745.3073 | 19.23 | yes | Prenol lipids | Tetrapyrroles and derivatives |
| 5603 | 731.2910 | 16.52 | no | Carboxylic acids and derivatives | Tetrapyrroles and derivatives |
| 5602 | 731.2910 | 16.57 | yes | Carboxylic acids and derivatives | Tetrapyrroles and derivatives |
| 5604 | 731.2910 | 15.18 | yes | Carboxylic acids and derivatives | Tetrapyrroles and derivatives |
| 5411 | 699.3013 | 19.44 | yes | Prenol lipids | Tetrapyrroles and derivatives |
| 5410 | 699.2651 | 19.44 | yes | Lignan glycosides | Tetrapyrroles and derivatives |
| 5492 | 713.2809 | 19.69 | yes | Prenol lipids | Tetrapyrroles and derivatives |
| 5277 | 681.2544 | 19.91 | yes | Diarylheptanoids | Benzene and substituted derivatives |
| 5045 | 645.2910 | 14.89 | no | Steroids and steroid derivatives | Tetrapyrroles and derivatives |
| 5331 | 687.3013 | 19.22 | yes | Prenol lipids | Pyrroles |
| 4903 | 627.2791 | 16.30 | yes | Steroids and steroid derivatives | Tetrapyrroles and derivatives |
| 5047 | 645.2912 | 16.31 | yes | Steroids and steroid derivatives | Tetrapyrroles and derivatives |
| 6190 | 837.4050 | 19.97 | no | Carboxylic acids and derivatives | Not classified |
| 4920 | 629.2964 | 19.48 | yes | Prenol lipids | Tetrapyrroles and derivatives |
| 5702 | 745.2698 | 19.20 | yes | Prenol lipids | Carboxylic acids and derivatives |
| 5703 | 745.2700 | 19.12 | yes | Prenol lipids | Tetrapyrroles and derivatives |
| 5706 | 745.2708 | 13.56 | yes | Prenol lipids | Carboxylic acids and derivatives |
| 5708 | 745.2708 | 13.19 | yes | Prenol lipids | Carboxylic acids and derivatives |
| 5705 | 745.2705 | 14.78 | no | Prenol lipids | Carboxylic acids and derivatives |
| 5707 | 745.2707 | 14.74 | yes | Prenol lipids | Carboxylic acids and derivatives |
| 5133 | 659.2703 | 13.76 | yes | Prenol lipids | Tetrapyrroles and derivatives |
| 5135 | 659.2705 | 13.37 | no | Prenol lipids | Tetrapyrroles and derivatives |
| 5802 | 759.2858 | 18.89 | yes | Fatty Acyls | Tetrapyrroles and derivatives |
| 5953 | 781.268 | 18.90 | yes | Stilbenes | Carboxylic acids and derivatives |
| 5132 | 659.2703 | 19.04 | yes | Lignan glycosides | Tetrapyrroles and derivatives |
| 5136 | 659.2708 | 12.68 | no | Lignan glycosides | Carboxylic acids and derivatives |


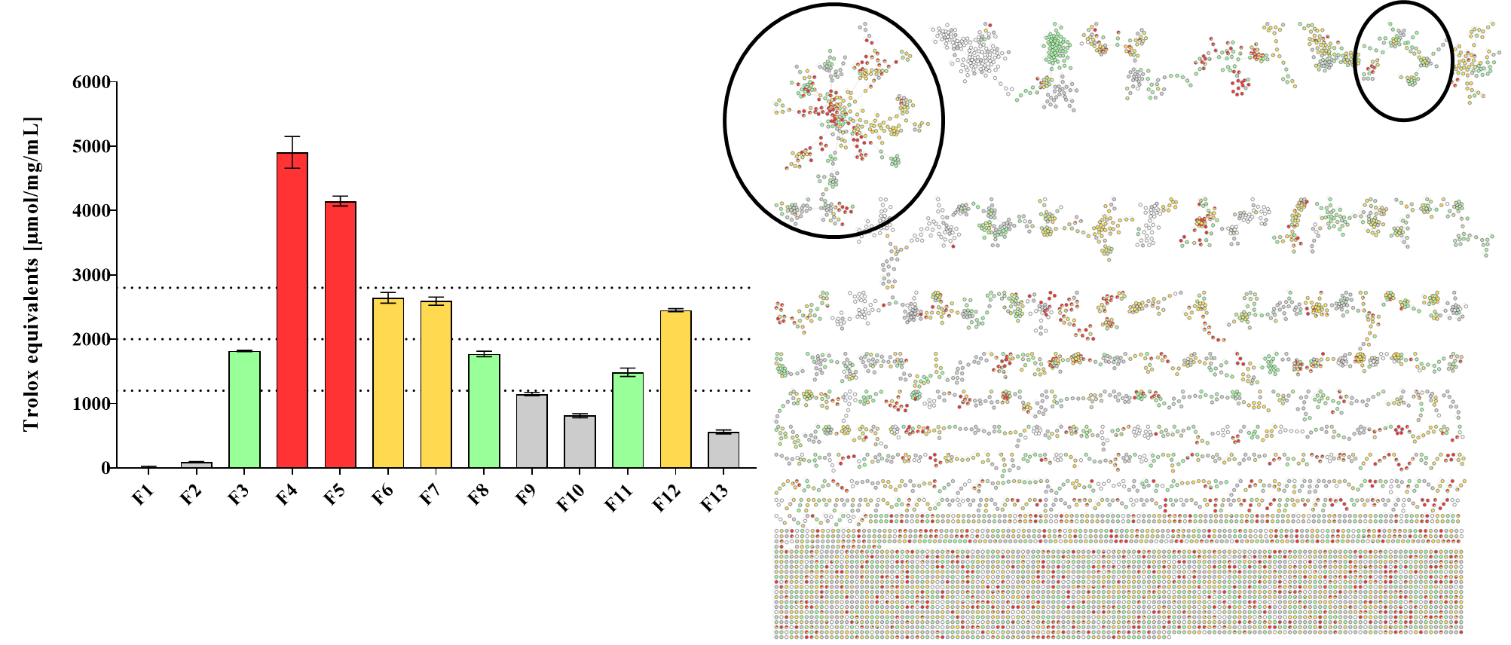


**Figure S9**. Informed feature-based molecular network of *S. officinalis* fractions, integrating bioactivity data from the FRAP assay. Highlighted is the cluster, which contains mainly nodes from the most active fractions and the phyllobilin cluster.

**Table S3.** Annotations for the most active FRAP nodes (colored in red) from the cluster (Figure S9)

| ID | Precursor mass [M+H]^+^ | Retention time [min] | CANOPUS annotation NPC class | CANOPUS annotation NPC subclass | CANOPUS annotation ClassyFire class |
| --- | --- | --- | --- | --- | --- |
| 385 | 191.1428 | 2.19 | Caryophyllane sesquiterpenoids | Sesquiterpenoids | Prenol lipids |
| 388 | 191.1428 | 4.17 | Apocarotenoids | Sesquiterpenoids | Prenol lipids |
| 391 | 191.1428 | 2.75 | Apocarotenoids | Sesquiterpenoids | Prenol lipids |
| 743 | 237.1118 | 1.37 | Cinnamic acids and derivatives | Phenylpropanoids (C6-C3) | Phenol ethers |
| 2123 | 366.1754 | 4.14 | Cyanogenic glycosides | Amino acid glycosides | Organooxygen compounds |
| 2124 | 366.1755 | 4.40 | Purine alkaloids | Pseudoalkaloids (transamidation) | Organooxygen compounds |
| 2220 | 374.1442 | 2.86 | Cyanogenic glycosides | Amino acid glycosides | Organooxygen compounds |
| 2281 | 381.1540 | 4.29 | Secoiridoid monoterpenoids | Monoterpenoids | Prenol lipids |
| 2354 | 388.1597 | 3.95 | Cyanogenic glycosides | Amino acid glycosides | Organooxygen compounds |
| 2374 | 390.2118 | 6.40 | Fatty acyl glycosides of mono- and disaccharides | Fatty acyl glycosides | Saccharolipids |
| 2409 | 395.1671 | 6.38 | Not applicable | Not applicable | Not applicable |
| 2440 | 398.2017 | 4.35 | Fatty acyl glycosides of mono- and disaccharides | Fatty acyl glycosides | Organooxygen compounds |
| 2444 | 399.1644 | 4.12 | Secoiridoid monoterpenoids | Apocarotenoids | Saccharolipids |
| 2445 | 399.1644 | 3.97 | Secoiridoid monoterpenoids | Apocarotenoids | Saccharolipids |
| 2446 | 399.1644 | 1.38 | Secoiridoid monoterpenoids | Monoterpenoids | Organooxygen compounds |
| 2447 | 399.1645 | 3.75 | Secoiridoid monoterpenoids | Apocarotenoids | Saccharolipids |
| 2448 | 399.1645 | 4.29 | Secoiridoid monoterpenoids | Apocarotenoids | Saccharolipids |
| 2450 | 399.1646 | 3.46 | Secoiridoid monoterpenoids | Monoterpenoids | Saccharolipids |
| 2474 | 401.1802 | 4.25 | Cinnamic acids and derivatives | Monoterpenoids | Organooxygen compounds |
| 2623 | 415.1570 | 2.91 | Fatty acyl glycosides of mono- and disaccharides | Fatty acyl glycosides | Saccharolipids |
| 2624 | 415.1570 | 4.98 | Fatty acyl glycosides of mono- and disaccharides | Fatty acyl glycosides | Saccharolipids |
| 2661 | 417.1749 | 4.15 | Secoiridoid monoterpenoids | Monoterpenoids | Saccharolipids |
| 2663 | 417.1750 | 4.30 | Secoiridoid monoterpenoids | Monoterpenoids | Organooxygen compounds |
| 2673 | 418.2066 | 4.25 | Secoiridoid monoterpenoids | Monoterpenoids | Prenol lipids |
| 2761 | 427.1566 | 3.87 | Purine alkaloids | Nucleosides | Carboxylic acids and derivatives |
| 2817 | 432.1860 | 3.52 | Cyanogenic glycosides | Amino acid glycosides | Organooxygen compounds |
| 2853 | 434.2016 | 3.97 | Cyanogenic glycosides | Amino acid glycosides | Organooxygen compounds |
| 2854 | 434.2016 | 4.15 | Fatty acyl glycosides of mono- and disaccharides | Apocarotenoids | Saccharolipids |
| 2855 | 434.2016 | 3.75 | Secoiridoid monoterpenoids | Monoterpenoids | Saccharolipids |
| 2856 | 434.2016 | 4.70 | Secoiridoid monoterpenoids | Apocarotenoids | Organooxygen compounds |
| 2857 | 434.2016 | 4.87 | Secoiridoid monoterpenoids | Monoterpenoids | Saccharolipids |
| 2859 | 434.2016 | 4.28 | Fatty acyl glycosides of mono- and disaccharides | Fatty acyl glycosides | Saccharolipids |
| 2861 | 434.2017 | 3.46 | Secoiridoid monoterpenoids | Monoterpenoids | Saccharolipids |
| 2889 | 436.2182 | 3.52 | Cyanogenic glycosides | Amino acid glycosides | Organooxygen compounds |
| 2917 | 439.1567 | 1.37 | Cinnamic acids and derivatives | Monoterpenoids | Organooxygen compounds |
| 2921 | 439.1570 | 4.50 | Secoiridoid monoterpenoids | Sesquiterpenoids | Organooxygen compounds |
| 2922 | 439.1570 | 4.17 | Fatty acyl glycosides of mono- and disaccharides | Fatty acyl glycosides | Saccharolipids |
| 2923 | 439.1570 | 4.29 | Fatty acyl glycosides of mono- and disaccharides | Fatty acyl glycosides | Saccharolipids |
| 2924 | 439.1571 | 4.70 | Megastigmanes | Apocarotenoids | Organooxygen compounds |
| 2925 | 439.1571 | 3.46 | Fatty acyl glycosides of mono- and disaccharides | Sesquiterpenoids | Organooxygen compounds |
| 2927 | 439.1575 | 3.75 | Megastigmanes | Apocarotenoids | Saccharolipids |
| 2954 | 441.1727 | 4.25 | Iridoids monoterpenoids | Monoterpenoids | Prenol lipids |
| 3011 | 446.2016 | 5.97 | Secoiridoid monoterpenoids | Monoterpenoids | Organooxygen compounds |
| 3075 | 451.1569 | 5.97 | Cinnamic acids and derivatives | Monoterpenoids | Organooxygen compounds |
| 3135 | 455.1306 | 1.36 | Not applicable | Not applicable | Not applicable |
| 3138 | 455.1311 | 4.86 | Not applicable | Not applicable | Not applicable |
| 3139 | 455.1312 | 4.29 | Prezizaane sesquiterpenoids | Sesquiterpenoids | Fatty Acyls |
| 3140 | 455.1316 | 3.48 | Megastigmanes | Apocarotenoids | Prenol lipids |
| 3145 | 455.1904 | 3.09 | Megastigmanes | Apocarotenoids | Prenol lipids |
| 3178 | 457.206 | 2.76 | Megastigmanes | Apocarotenoids | Prenol lipids |
| 3291 | 467.1157 | 2.39 | Secoiridoid monoterpenoids | Monoterpenoids | Organooxygen compounds |
| 3292 | 467.1516 | 1.98 | Aminosugars | Aminosugars and aminoglycosides | Organooxygen compounds |
| 3400 | 475.2168 | 2.76 | Secoiridoid monoterpenoids | Apocarotenoids | Saccharolipids |
| 3406 | 476.1757 | 1.79 | Iridoids monoterpenoids | Monoterpenoids | Prenol lipids |
| 3476 | 481.131 | 1.81 | Secoiridoid monoterpenoids | Monoterpenoids | Organooxygen compounds |
| 3493 | 483.0896 | 2.38 | Not applicable | Not applicable | Not applicable |
| 3608 | 492.2429 | 2.76 | Megastigmanes | Apocarotenoids | Prenol lipids |
| 3633 | 495.1467 | 4.37 | Simple coumarins | Ornithine alkaloids | Phenol ethers |
| 3637 | 495.1826 | 3.08 | Megastigmanes | Apocarotenoids | Saccharolipids |
| 3638 | 495.1830 | 2.61 | Megastigmanes | Apocarotenoids | Prenol lipids |
| 3639 | 495.1830 | 2.75 | Fatty acyl glycosides of mono- and disaccharides | Sesquiterpenoids | Saccharolipids |
| 3640 | 495.1830 | 3.85 | Secoiridoid monoterpenoids | Monoterpenoids | Organooxygen compounds |
| 3654 | 497.1050 | 1.80 | Simple coumarins | Small peptides | Benzene and substituted derivatives |
| 3661 | 497.1774 | 1.64 | Cyanogenic glycosides | Amino acid glycosides | Cinnamic acids and derivatives |
| 3662 | 497.1984 | 2.76 | Megastigmanes | Apocarotenoids | Saccharolipids |
| 3663 | 497.1990 | 3.73 | Megastigmanes | Sesquiterpenoids | Prenol lipids |
| 3824 | 511.1209 | 4.37 | Not applicable | Not applicable | Not applicable |
| 3830 | 511.1578 | 2.74 | Not applicable | Not applicable | Not applicable |
| 3843 | 513.1732 | 2.76 | Not applicable | Not applicable | Not applicable |
| 3916 | 521.2014 | 2.04 | Furanoid lignans | Lignans | Lignan glycosides |
| 3970 | 526.2277 | 2.61 | Furofuranoid lignans | Lignans | Lignan glycosides |
| 4007 | 531.1831 | 2.62 | Arylnaphthalene and aryltetralin lignans | Lignans | Lignan glycosides |
| 4259 | 556.2380 | 2.04 | Purine alkaloids | Oligopeptides | Phenol ethers |
| 4260 | 556.2380 | 2.66 | Purine nucleosides | Nucleosides | Imidazopyrimidines |
| 4460 | 578.2074 | 3.54 | Secoiridoid monoterpenoids | Monoterpenoids | Saccharolipids |
| 4503 | 583.1628 | 3.54 | Pterocarpan | Monoterpenoids | Organooxygen compounds |
| 4708 | 607.2012 | 2.73 | Secoiridoid monoterpenoids | Monoterpenoids | Prenol lipids |
| 4776 | 612.2285 | 2.89 | Terpenoid tetrahydroisoquinoline alkaloids | Tyrosine alkaloids | Organooxygen compounds |
| 4828 | 617.1835 | 3.30 | Pterocarpan | Isoflavonoids | Isoflavonoids |
| 4990 | 640.2231 | 4.09 | Linear peptides | Oligopeptides | Carboxylic acids and derivatives |
| 4992 | 640.2234 | 3.56 | Isoquinoline alkaloids | Tyrosine alkaloids | Carboxylic acids and derivatives |
| 5015 | 642.2387 | 2.75 | Carboline alkaloids | Tryptophan alkaloids | Carboxylic acids and derivatives |
| 5016 | 642.2388 | 2.57 | Carboline alkaloids | Tryptophan alkaloids | Indoles and derivatives |
| 5054 | 647.1939 | 2.57 | Pterocarpan | Isoflavonoids | Isoflavonoids |
| 5057 | 647.1941 | 2.75 | Pterocarpan | Isoflavonoids | Isoflavonoids |


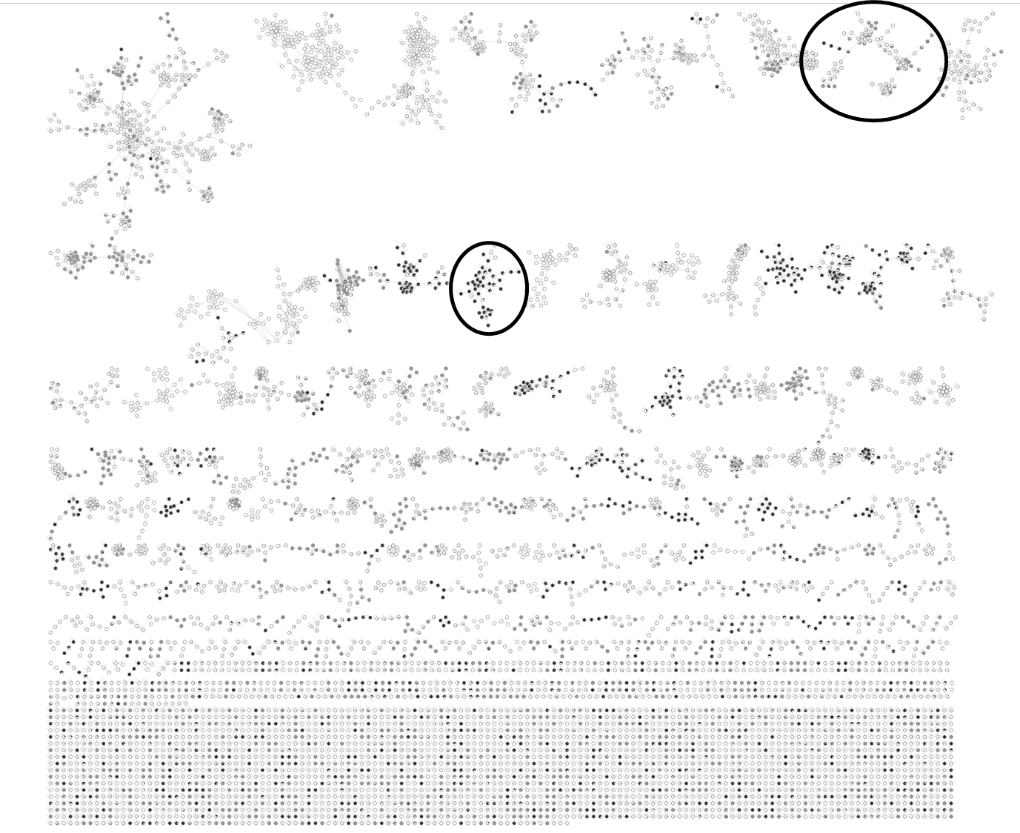


**Figure S10.** Informed feature-based molecular network of *S. officinalis* fractions, integrating bioactivity data from the cellular ROS assay. Highlighted is the cluster, which contains mainly nodes from the most active fractions and the phyllobilin cluster.


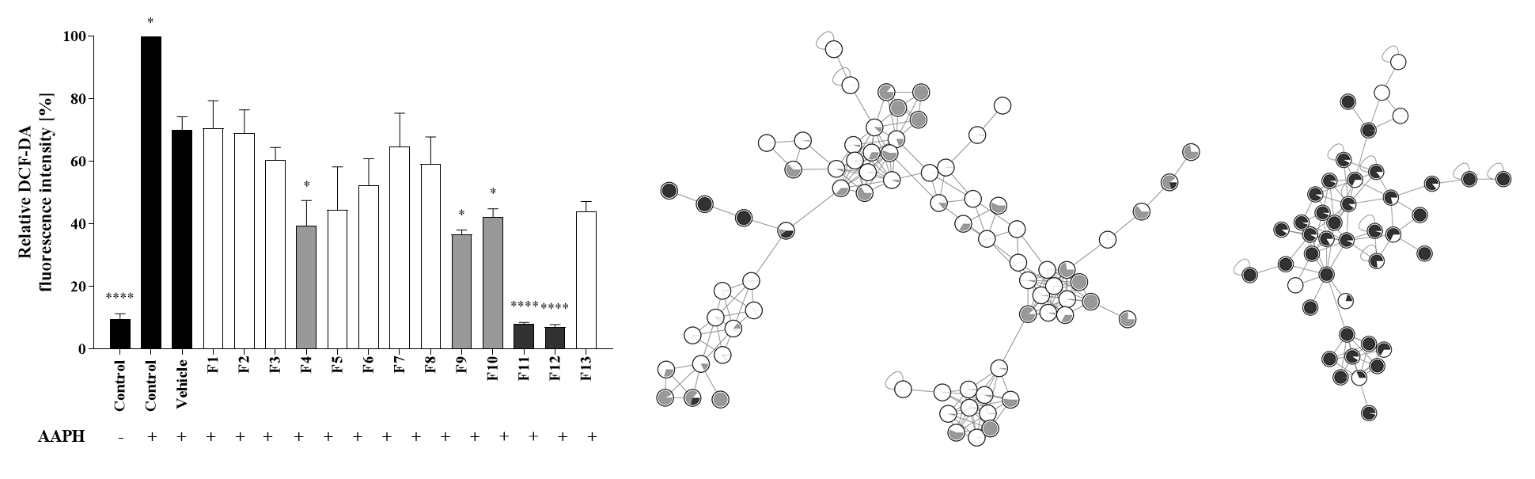


**Figure S11.** Informed feature-based molecular networking: incorporating the bioactivity measured with a cellular ROS assay into the molecular network of *S. officinalis* fractions. Left shows the Relative DCF-DA fluorescence intensity of all fractions. The bars are colored empirically based on activity. White represents inactive, light grey active, and dark grey very active fractions. Each node is depicted as a pie chart consisting of the peak intensity of the fraction the masses were found + the designated color of the fraction based on the ROS assay results. In the middle is the phyllobilin cluster and on the right side is a cluster with the most active compounds.

**Table S4.** Annotations for the most active ROS assay nodes

| ID | Precursor mass [M+H]^+^ | Retention time [min] | CANOPUS annotation NPC class | CANOPUS annotation NPC subclass | CANOPUS annotation ClassyFire class |
| --- | --- | --- | --- | --- | --- |
| 1954 | 347.2212 | 21.60 | Abietane diterpenoids | Diterpenoids | Prenol lipids |
| 2491 | 402.2632 | 16.91 | Abietane diterpenoids | Diterpenoids | Prenol lipids |
| 2492 | 402.2633 | 17.30 | Abietane diterpenoids | Diterpenoids | Carboxylic acids and derivatives |
| 2638 | 416.2425 | 19.77 | Simple coumarins | Coumarins | Carboxylic acids and derivatives |
| 2639 | 416.2426 | 20.12 | Aminoacids | Small peptides | Carboxylic acids and derivatives |
| 2780 | 428.2789 | 20.08 | Isoquinoline alkaloids | Tyrosine alkaloids | Not classified |
| 2781 | 428.2790 | 19.20 | Abietane diterpenoids | Diterpenoids | Carboxylic acids and derivatives |
| 3362 | 472.2683 | 19.32 | Open-chain polyketides | Macrolides | Not classified |
| 5026 | 643.2532 | 20.84 | Depsides and depsidones | Diterpenoids | Phenols |
| 5217 | 674.3678 | 20.86 | Polyene macrolides | Macrolides | Not classified |
| 5218 | 674.3678 | 20.61 | Polyene macrolides | Macrolides | Carboxylic acids and derivatives |
| 5219 | 674.3679 | 20.43 | Polyene macrolides | Macrolides | Carboxylic acids and derivatives |
| 5230 | 675.3885 | 21.85 | Abietane diterpenoids | Diterpenoids | Prenol lipids |
| 5231 | 675.3887 | 21.60 | Abeoabietane diterpenoids | Diterpenoids | Not classified |
| 5236 | 676.3836 | 21.04 | Open-chain polyketides | Macrolides | Not classified |
| 5237 | 676.3839 | 21.35 | Polyene macrolides | Macrolides | Not classified |
| 5253 | 678.3993 | 21.54 | Polyene macrolides | Macrolides | Macrolides and analogues |
| 5254 | 678.3995 | 21.35 | Abietane diterpenoids | Diterpenoids | Prenol lipids |
| 5283 | 681.3397 | 21.50 | Depsipeptides | Oligopeptides | Carboxylic acids and derivatives |
| 5297 | 683.3543 | 21.35 | Depsipeptides | Oligopeptides | Carboxylic acids and derivatives |
| 5353 | 692.4141 | 21.37 | Open-chain polyketides | Macrolides | Macrolides and analogues |
| 5354 | 692.4150 | 21.61 | Spirotetronate macrolides | Diterpenoids | Phenols |
| 5382 | 697.3110 | 21.49 | Open-chain polyketides | Macrolides | Carboxylic acids and derivatives |
| 5385 | 697.3703 | 21.61 | Polyene macrolides | Macrolides | Macrolides and analogues |
| 5386 | 697.3707 | 22.31 | Cyclic peptides | Oligopeptides | Carboxylic acids and derivatives |
| 5413 | 699.3272 | 21.35 | Cyclic peptides | Oligopeptides | Carboxylic acids and derivatives |
| 5415 | 699.3517 | 20.61 | Depsipeptides | Oligopeptides | Carboxylic acids and derivatives |
| 5442 | 704.4151 | 21.35 | Open-chain polyketides | Oligopeptides | Carboxylic acids and derivatives |
| 5451 | 706.3940 | 21.13 | Depsipeptides | Oligopeptides | Carboxylic acids and derivatives |
| 5453 | 706.4304 | 21.60 | Open-chain polyketides | Macrolides | Carboxylic acids and derivatives |
| 5471 | 711.3498 | 21.12 | Open-chain polyketides | Macrolides | Not classified |
| 5493 | 713.3442 | 21.59 | Not applicable | Not applicable | Not applicable |
| 5542 | 718.4307 | 21.59 | Open-chain polyketides | Macrolides | Not classified |
| 6048 | 803.5190 | 16.88 | Cardenolides | Steroids | Steroids and steroid derivatives |
| 6552 | 1006.5669 | 21.35 | Not applicable | Not applicable | Not applicable |
| 6558 | 1020.5825 | 21.61 | Not applicable | Not applicable | Not applicable |
| 6616 | 1339.6872 | 21.49 | Not applicable | Not applicable | Not applicable |
| 6617 | 1343.7194 | 21.35 | Not applicable | Not applicable | Not applicable |
| 6622 | 1371.7512 | 21.59 | Not applicable | Not applicable | Not applicable |

**Table S5**. Structural network analysis of the phyllobilin cluster in *S. officinalis* extracts with key fragmentations that were used to postulate structures.

| ID | Precursor mass experimental [M+H]^+^ | Postulated Structure | Precursor mass calculated  [M+H]^+^ | Mass difference [ppm] | MS^2^ feature (-MeOH) and mass difference calculated vs. experimental | MS^2^ feature (- ring A) and mass difference calculated vs. experimental | MS^2^ feature (ring A fragment) and mass difference calculated vs. experimental | MS^2^ feature (- ring D) and mass difference calculated vs. experimental | MS^2^ feature (ring D fragment) and mass difference calculated vs. experimental |
| --- | --- | --- | --- | --- | --- | --- | --- | --- | --- |
| 5586 | 729.2746 | *Sao*-PxB (4) | 729.2766 | -2.7 | 697.2498 (-0.9 ppm) | 490.1959 (-2.9 ppm) | 252.0861 (-2.0 ppm) |  |  |
| 5587 | 729.2753 | *Sao*-PxB (4) | 729.2766 | -1.8 | 697.2504 (0 ppm) | 490.1979 (1.2 ppm) | 252.0874 (3.2 ppm) |  |  |
| 5588 | 729.2753 | *Sao*-PxB (4) | 729.2766 | -1.8 | 697.2497 (-1.0 ppm) | 490.1995 (4.5 ppm) | 252.0866 (7.9 ppm) |  |  |
| 5589 | 729.2754 | *Sao*-PxB (4) | 729.2766 | -1.6 | 697.2496 (-1.1 ppm) | 490.1971 (-0.4 ppm) | 252.0856 (-4.0 ppm) |  |  |
| 5590 | 729.2754 | *Sao*-PxB (4) | 729.2766 | -1.6 | 697.2501 (-0.4 ppm) | 490.1955 (-3.7 ppm) | 252.0867 (0.4 ppm) |  |  |
| 5591 | 729.2756 | *Sao*-PxB (4) | 729.2766 | -1.4 | 697.2504 (-0.7 ppm) | 490.1973 (1.4 ppm) | 252.0869 (1.2 ppm) |  |  |
| 5592 | 729.2757 | *Sao*-PxB (4) | 729.2766 | -1.2 | 697.2502 (-0.3 ppm) | 490.1973 (-1.8 ppm) | 252.0876 (4.0 ppm) |  |  |
| 5045 | 645.2910 | *Ep*-PleB-6 (5) | 645.2919 | -1.4 | 613.2654 (-0.3 ppm) | 492.2137 (1.6 ppm) | 166.0865 (1.2 ppm) | Not detected | 136.0754 (-1.5 ppm) |
| 5046 | 645.2911 | *Ep*-PleB-6  (5) | 645.2919 | -1.2 | 613.2655 (-0.2 ppm) | 492.2122 (-1.4 ppm) | 166.0863 (0 ppm) | 522.2234 (0 ppm) | 136.0756 (0 ppm) |
| 5601 | 731.2909 | *Sao*-PleB (6) | 731.2923 | -1.9 | 699.2652 (-1.3 ppm) | 492.2099 (-6.1 ppm) | Not detected | 608.2239 (0.8 ppm) | Not detected |
| 5602 | 731.2910 | *Sao*-PleB (6) | 731.2923 | -1.8 | 699.2650 (-1.6 ppm) | 492.2123 (-1.2 ppm) | 252.0874 (3.2 ppm) | 608.2224 (-2.5 ppm) | 136.0757 (0 ppm) |
| 5603 | 731.2910 | *Sao*-PleB (6) | 731.2923 | -1.8 | 699.2659 (-0.3 ppm) | Not detected | 252.0869 (1.2 ppm) | Not detected | 136.0753 (-2.9 ppm) |
| 5604 | 731.2910 | *Sao*-PleB (6) | 731.2923 | -1.8 | 699.2654 (-1.0 ppm) | Not detected | 252.0870 (1.6 ppm) | 608.2235 (-0.7 ppm) | 136.0756 (-0.7 ppm) |
| 5005 | 641.2606 | *Ep*-PrB-6 (1) | 641.2606 | 0 | 609.2344 (-0.3 ppm) | 488.1816 (0 ppm) | 166.0863 (0.6 ppm) |  |  |
| 5573 | 727.2597 | *Sao*-PrB (2) | 727.2597 | -1.8 | 695.2326 (-3.2 ppm) | 488.1804 (-2.5 ppm) | Not detected |  |  |
| 5132 | 659.2703 | *Ep*-PleB-6 + Carbonyl (7) | 659.2712 | -1.4 | 627.2440 (-1.4 ppm) | 506.1927 (1.0 ppm) | 166.0865 (1.2 ppm) | Not detected | 136.0760 (2.9 ppm) |
| 5133 | 659.2703 | *Ep*-PleB-6 + Carbonyl (7) | 659.2712 | -1.4 | 627.2446 (-0.5 ppm) | Not detected | 166.0862 (-0.6 ppm) | Not detected | 136.0756 (0 ppm) |
| 5135 | 659.2703 | *Ep*-PleB-6 + Carbonyl (7) | 659.2712 | -1.4 | 627.2444 (-0.8 ppm) | 506.1937 (3.0 ppm) | 166.0863 (-3.6 ppm) | Not detected | 136.0761 (3.7 ppm) |
| 5703 | 745.2700 | *Sao*-PleB + Carbonyl (8) | 745.2715 | -2 | 713.2457 (0.6 ppm) | Not detected | 252.0863 (-1.2 ppm) | Not detected | 136.0758 (1.5 ppm) |
| 5705 | 745.2705 | *Sao*-PleB + Carbonyl (8) | 745.2715 | -1.3 | 713.2447 (-0.8 ppm) | Not detected | 252.0867 (0.4 ppm) | Not detected | 136.0761 (3.7 ppm) |
| 5706 | 745.2708 | *Sao*-PleB + Carbonyl (8) | 745.2715 | -0.9 | 713.2446 (-0.8 ppm) | Not detected | 252.0872 (2.4 ppm) | Not detected | 136.0754 (-1.5 ppm) |
| 5707 | 745.2707 | *Sao*-PleB + Carbonyl (8) | 745.2715 | -1.1 | 713.2446 (-0.8 ppm) | 506.1926 (0.8 ppm) | 252.0873 (2.8 ppm) | Not detected | 136.0757 (0.7 ppm) |
| 5708 | 745.2708 | *Sao*-PleB + Carbonyl (8) | 745.2715 | -0.9 | 713.2458 (0.7 ppm) | Not detected | 252.0878 (4.8 ppm) | Not detected | 136.0756 (-0.7 ppm) |


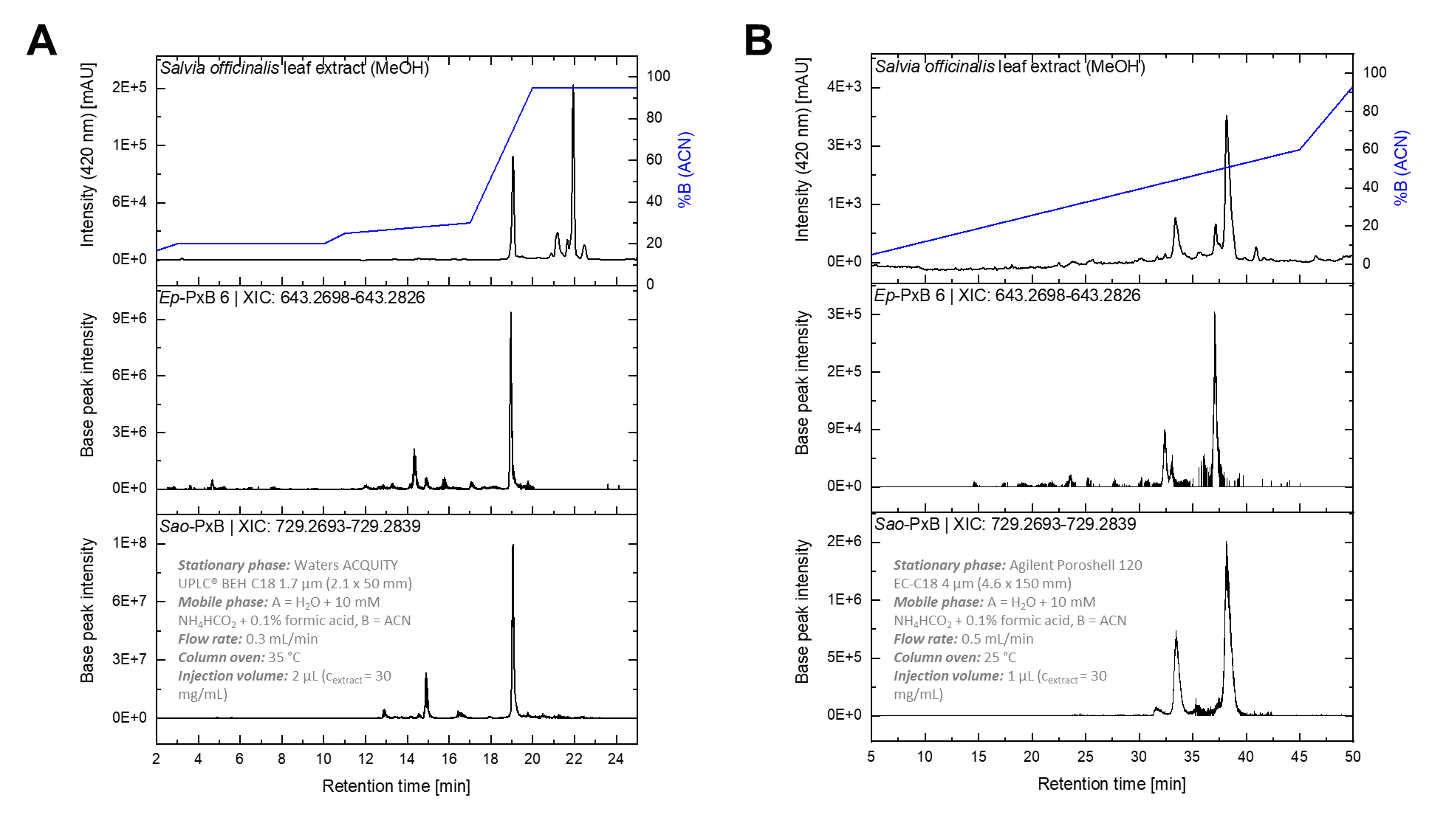


**Figure S12.** Chromatograms of the methanolic *S. officinalis* extract at 420 nm, the extracted ion chromatograms of isolated *Ep*-PxB-6 (XIC: 643.2698-643.2826 m/z), and *Sao*-PxB (XIC: 729.2693-729.2839 m/z) analyzed on a Vanquish system (Thermo Scientific, Waltham, Massachusetts, USA) consisting of a quaternary pump, an auto-sampler, a column oven, and a variable wavelength detector connected to a Thermo Scientific Exploris 120 Orbitrap HRMS unit: A) depicts the chromatograms recorded with the method which was used to analyze all plant fractions: separation was carried out on a Waters ACQUITY UPLC BEH C18 column (2.1 mm x 50 mm, particle size = 1.7 µm) (Waters Corporation, Milford, Massachusetts, USA) protected by a Phenomenex SecurityGuard ULTRA guard cartridge system (i.e., a UHPLC C18 pre-column) (Phenomenex, Aschaffenburg, Germany). The mobile phase comprised ammonium formate (c = 10 mM) in water with 0.1% formic acid (A) and acetonitrile (B). The applied gradient was as follows: 0 min, 10% B; 3 min 20% B; 10 min, 20% B; 11 min, 25% B; 17 min, 30% B; 20 min, 95% B; 26 min, 95% B. Finally, the column was re-equilibrated with the original solvent composition for 12 minutes. The flow rate, column oven temperature, auto-sampler temperature, and injection volume were adjusted to 0.3 mL/min, 35 °C, 20 °C, and 2 µL, respectively. The detection wavelength was set to 420 nm. For MS parameters see Text S2.

*Ep*-PxB-6 has a retention time of 18.94 min and *Sao*-PxB has a retention time of 19.04 min. The chromatogram of the methanolic *S. officinalis* extract at 420 nm does not distinguish between these two PxBs.

B) shows the chromatograms with a different analytical method: separation was carried out on a Agilent Poroshell 120 EC-C18 column (4.6 mm x 150 mm, particle size = 4 µm) (Agilent, Santa Clara, USA) protected by a Phenomenex SecurityGuard guard C18 cartridge system (Phenomenex, Aschaffenburg, Germany). The mobile phase comprised ammonium formate (c = 10 mM) in water with 0.1% formic acid (A) and acetonitrile (B). The applied gradient was as follows: 0 min, 5% B; 45 min 60% B; 51 min, 100% B; 53 min, 100% B; the column was re-equilibrated with the original solvent composition for 7 minutes. The flow rate, column oven temperature, auto-sampler temperature, and injection volume were adjusted to 0.5 mL/min, 25 °C, 20 °C, and 1 µL, respectively. The detection wavelength was set to 420 nm. Mass parameters: heated-ESI source, static spray voltage (positive: 4500 V), sheath gas (N2): 35 arbitrary units, auxiliary gas (N2): 15 arbitrary units, sweep gas (N2): 1 arbitrary unit. Temperature of the ion transfer tube and vaporizer was adjusted to 370 and 420 °C, respectively. For other MS parameters see Text S2.

*Ep*-PxB-6 has a retention time of 37.03 min and *Sao*-PxB has a retention time of 38.23 min. The chromatogram of the methanolic S. officinalis extract at 420 nm shows multiple distinct peaks corresponding to both PxBs, which proves that *Ep*-PxB-6 is not an in-source fragment.


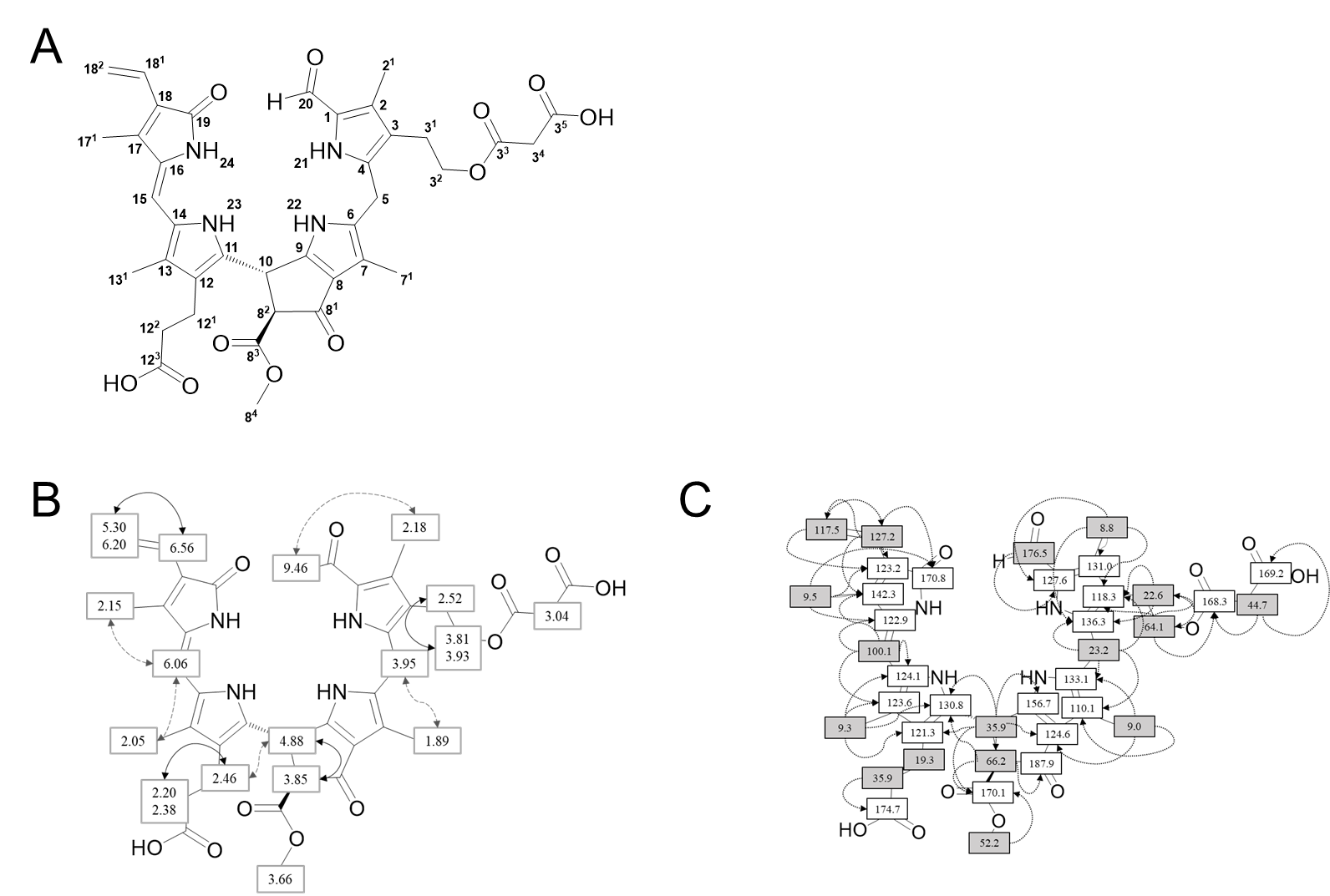


**Figure S13.** Summary of the 1D- and 2D- NMR experiments: A) depicts the structure of *Sao*-PxB with the numbering of the protons. B) shows the chemical shifts of the ^1^H-NMR in boxes, black arrows represent ^1^H-COSY correlations, whereas dashed grey arrows visualize NOESY correlations. C) shows the chemical shift of the ^13^C-NMR signals in boxes, which were assigned based on HSQC data (shaded boxes) or through HMBC correlations (dashed grey arrows).


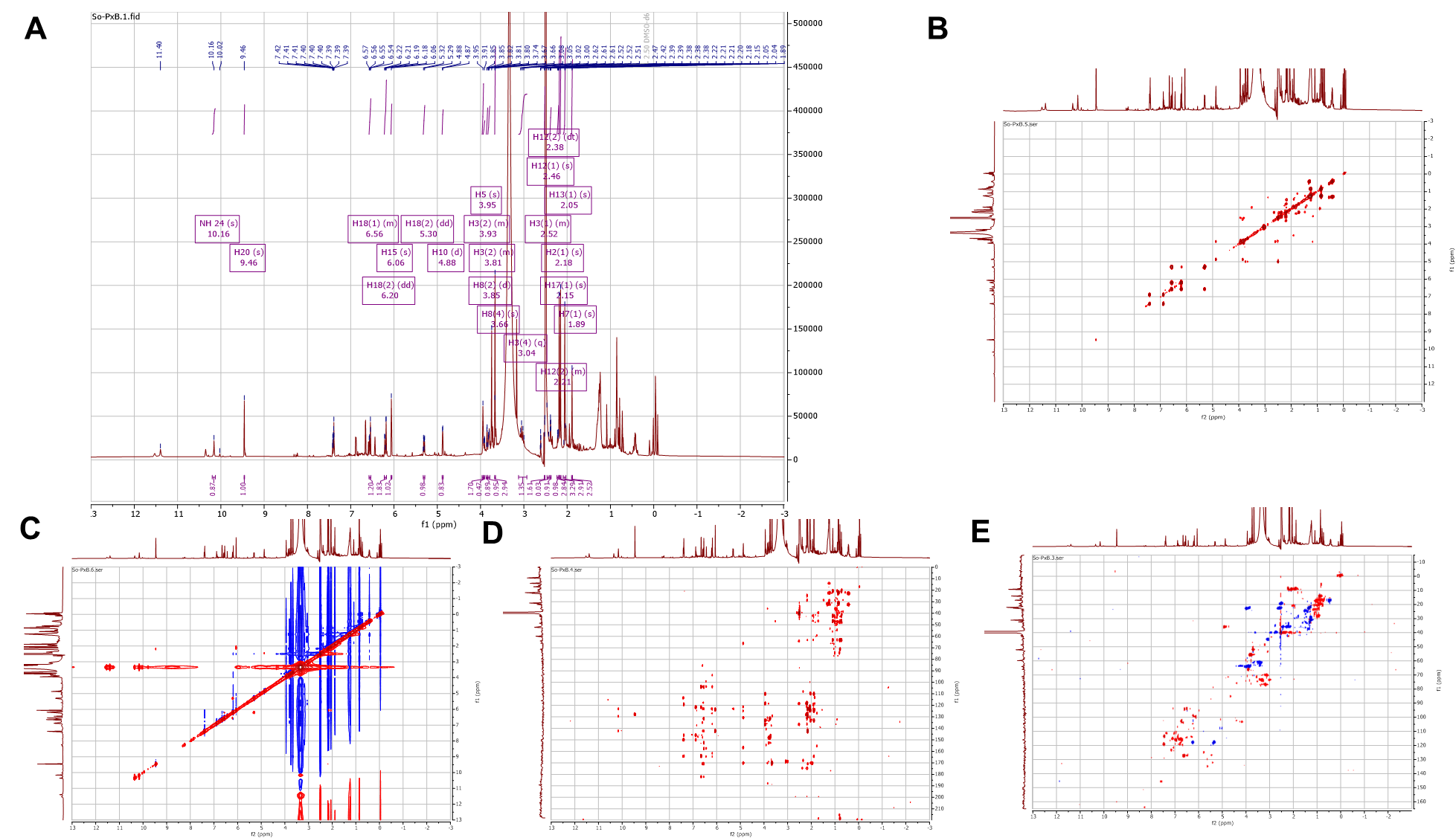


**Figure S14.** 1D and 2D NMR-spectra with assigned signals of *Sao*-PxB in d6-DMSO A) ^1^H-NMR spectrum, B) ^1^H,^1^H-COSY spectrum, C) ^1^H,^1^H-NOESY spectrum, D) ^1^H,^13^C-HMBC spectrum, and E) ^1^H,^13^C-HSQC spectrum.

**Table S6.** Overview of phyllobilins for which MS² reference spectra were uploaded to the GNPS public spectral library.

| **Name** | **Precursor mass [M+H]^+^** | **Retention time [min]** | **Molecular formula** | **SMILES** |
| --- | --- | --- | --- | --- |
| *Ep*-PxB-1 | 925.333 | 5.67 | C44H52N4O18 | O=C1C2=C(NC(CC3=C(C(C)=C(N3)C([H])=O)CCOC4OC(C(C(C4O)O)O)COC(CC(O)=O)=O)=C2C)[C@H]([C@@H]1C(OC)=O)C5=C(C(C)=C(N5)C=C(NC6=O)C(C)=C6C(CO)O)CCC(O)=O |
| *Ep*-PxB-2 | 839.333 | 5.20 | C41H50N4O15 | O=C1C2=C(NC(CC3=C(C(C)=C(N3)C([H])=O)CCOC4OC(C(C(C4O)O)O)CO)=C2C)[C@H]([C@@H]1C(OC)=O)C5=C(C(C)=C(N5)C=C(NC6=O)C(C)=C6C(CO)O)CCC(O)=O |
| *Ep*-PxB-3 | 677.279 | 6.34 | C35H40N4O10 | O=C1C2=C(NC(CC3=C(C(C)=C(N3)C([H])=O)CCO)=C2C)[C@H]([C@@H]1C(OC)=O)C4=C(C(C)=C(N4)C=C(NC5=O)C(C)=C5C(CO)O)CCC(O)=O |
| *Ep*-PxB-4 | 891.327 | 16.19 | C44H50N4O16 | O=C1C2=C(NC(CC3=C(C(C)=C(N3)C([H])=O)CCOC4OC(C(C(C4O)O)O)COC(CC(O)=O)=O)=C2C)[C@H]([C@@H]1C(OC)=O)C5=C(C(C)=C(N5)C=C(NC6=O)C(C)=C6C=C)CCC(O)=O |
| *Ep*-PxB-5 | 805.327 | 9.65 | C41H48N4O13 | O=C1C2=C(NC(CC3=C(C(C)=C(N3)C([H])=O)CCOC4OC(C(C(C4O)O)O)CO)=C2C)[C@H]([C@@H]1C(OC)=O)C5=C(C(C)=C(N5)C=C(NC6=O)C(C)=C6C=C)CCC(O)=O |
| *Ep*-PxB-6 | 643.275 | 19.04 | C35H38N4O8 | O=C1C2=C([C@@H](C3=C(CCC(O)=O)C(C)=C(C=C(C(C)=C4C=C)NC4=O)N3)[C@@H]1C(OC)=O)NC(CC5=C(CCO)C(C)=C(C([H])=O)N5)=C2C |
| *Sao*-PxB | 729.275 | 19.18 | C38H40N4O11 | O=C1C2=C([C@@H](C3=C(CCC(O)=O)C(C)=C(C=C(C(C)=C4C=C)NC4=O)N3)[C@@H]1C(OC)=O)NC(CC5=C(CCOC(CC(O)=O)=O)C(C)=C(C([H])=O)N5)=C2C |

**References**

1. Zuo, Z., et al., *MS2Planner: improved fragmentation spectra coverage in untargeted mass spectrometry by iterative optimized data acquisition.* Bioinformatics, 2021. **37**(Supplement_1): p. i231-i236.

2. Chambers, M.C., et al., *A cross-platform toolkit for mass spectrometry and proteomics.* Nature Biotechnology, 2012. **30**(10): p. 918-920.

3. Schmid, R., et al., *Integrative analysis of multimodal mass spectrometry data in MZmine 3.* Nature Biotechnology, 2023. **41**(4): p. 447-449.

4. Schmid, R., et al., *Ion identity molecular networking for mass spectrometry-based metabolomics in the GNPS environment.* Nature Communications, 2021. **12**(1): p. 3832.

5. Wohlgemuth, G., et al., *SPLASH, a hashed identifier for mass spectra.* Nature Biotechnology, 2016. **34**(11): p. 1099-1101.

6. Horai, H., et al., *MassBank: a public repository for sharing mass spectral data for life sciences.* Journal of Mass Spectrometry, 2010. **45**(7): p. 703-714.

7. Mohimani, H., et al., *Dereplication of microbial metabolites through database search of mass spectra.* Nature Communications, 2018. **9**(1): p. 4035.

8. Shannon, P., et al., *Cytoscape: a software environment for integrated models of biomolecular interaction networks.* Genome Res, 2003. **13**(11): p. 2498-504.

9. Dührkop, K., et al., *SIRIUS 4: a rapid tool for turning tandem mass spectra into metabolite structure information.* Nature Methods, 2019. **16**(4): p. 299-302.

10. Ludwig, M., et al., *Database-independent molecular formula annotation using Gibbs sampling through ZODIAC.* Nature Machine Intelligence, 2020. **2**(10): p. 629-641.

11. Dührkop, K., et al., *Searching molecular structure databases with tandem mass spectra using CSI:FingerID.* Proceedings of the National Academy of Sciences, 2015. **112**(41): p. 12580-12585.

12. Dührkop, K., et al., *Systematic classification of unknown metabolites using high-resolution fragmentation mass spectra.* Nature Biotechnology, 2021. **39**(4): p. 462-471.

13. Rutz, A., et al., *Taxonomically Informed Scoring Enhances Confidence in Natural Products Annotation.* Frontiers in Plant Science, 2019. **Volume 10 - 2019**.

14. Djoumbou Feunang, Y., et al., *ClassyFire: automated chemical classification with a comprehensive, computable taxonomy.* Journal of Cheminformatics, 2016. **8**(1): p. 61.

15. Kim, H.W., et al., *NPClassifier: A Deep Neural Network-Based Structural Classification Tool for Natural Products.* Journal of Natural Products, 2021. **84**(11): p. 2795-2807.

16. Hammerle, F., et al., *Feature-Based Molecular Networking—An Exciting Tool to Spot Species of the Genus Cortinarius with Hidden Photosensitizers.* Metabolites, 2021. **11**(11): p. 791.

17. Hammerle, F., et al., *Highlighting the Phototherapeutical Potential of Fungal Pigments in Various Fruiting Body Extracts with Informed Feature-Based Molecular Networking.* Microbial Ecology, 2023. **86**(3): p. 1972-1992.

18. Moser, S., et al., *A yellow chlorophyll catabolite is a pigment of the fall colours.* Photochemical & Photobiological Sciences, 2008. **7**(12): p. 1577-1581.

19. Karg, C.A., et al., *A Chlorophyll-Derived Phylloxanthobilin Is a Potent Antioxidant That Modulates Immunometabolism in Human PBMC.* Antioxidants, 2022. **11**(10): p. 2056.
